# Using the Jigsaw Teaching Method to Enhance Internal Medicine Residents' Knowledge and Attitudes in Managing Geriatric Women's Health

**DOI:** 10.15766/mep_2374-8265.11003

**Published:** 2020-10-23

**Authors:** Patricia Ng, Kimberly Kranz, Ruth Abeles, Danielle Schwartz, Susan Lane

**Affiliations:** 1 Assistant Professor of Clinical Medicine, Department of Medicine, Renaissance School of Medicine at Stony Brook University; 2 Assistant Clinical Professor, Department of Medicine, University of California, San Diego; 3 Clinical Instructor, Department of Medicine, Mount Sinai Hospital; 4 Professor, Department of Medicine, Renaissance School of Medicine at Stony Brook University

**Keywords:** Geriatrics, Women's Health, Jigsaw Teaching Method, Ambulatory Education, Menopause, Urinary Incontinence, Abnormal Uterine Bleeding, Uterine Hemorrhage, Osteoporosis, Editor's Choice

## Abstract

**Introduction:**

Although studies surveying internal medicine (IM) residency program directors identify geriatric women's health as an essential curriculum topic, there are limited published women's health curricula for IM residents. Our IM residency program performed a needs assessment, which revealed that the majority of residents were unsatisfied with our current curricula and most were not confident managing geriatric women's health. We developed and assessed a structured curriculum to improve IM residents’ knowledge and confidence in addressing geriatric women's health.

**Methods:**

This 2-hour interactive workshop used the jigsaw teaching method (a cooperative learning strategy where peers deliver specific content in teams) to teach 84 categorical IM residents of all PGY levels about the diagnosis and management of menopause, osteoporosis, urinary incontinence, and abnormal uterine bleeding. Participants completed a pretest and immediate posttest to assess knowledge and confidence about the targeted topics. We compared baseline and postworkshop responses using chi-square and Wilcoxon signed rank tests.

**Results:**

Seventy-four (88%) IM residents completed the pretest, and 62 (74%) completed the posttest. Mean knowledge scores improved from 51% to 69% (*p* < .0001). Residents who reported feeling somewhat confident or confident in addressing women's health topics increased from 14% to 44% (*p* < .0001). The majority were satisfied or very satisfied with the workshop (94%) and requested additional women's health education (92%).

**Discussion:**

Our results suggest that workshops using the jigsaw teaching method can effectively increase IM resident knowledge and confidence in managing geriatric women's health.

## Educational Objectives

By the end of this activity, learners will be able to:

1. Diagnose abnormal uterine bleeding (AUB) through history, physical exam features, and diagnostic testing.
2. List potential etiologies for AUB using the International Federation of Gynecology and Obstetrics classification system.
3. List risk factors for endometrial carcinoma.
4. Describe treatment options for AUB.
5. List risk factors for osteoporosis.
6. Describe screening guidelines for osteoporosis.
7. Interpret bone mineral density test results and diagnose osteoporosis.
8. Describe pharmacological and nonpharmacological options to treat osteoporosis.
9. Identify clinical presentation of menopause.
10. Describe hormonal and nonhormonal treatment options for menopausal symptoms.
11. Describe the clinical presentation and workup of urinary incontinence (UI).
12. Describe nonpharmacologic, pharmacologic, and surgical options for treating UI.

## Introduction

Despite the need to provide gender- and age-specific care in primary care settings, studies have shown significant deficiencies in women's health knowledge, confidence, and training amongst internal medicine (IM) residency programs.^1,2^ Current literature shows that there are knowledge gaps in several women's health topics, with more recent studies suggesting a particular need to address geriatric women's health. In 2019, Kling and colleagues surveyed residents in IM, family medicine, and obstetrics and gynecology US residency programs and found that up to 50% of IM trainees did not feel prepared to manage menopause and over a quarter of the IM respondents had not had any didactics about menopause during residency.^3^ While 24% of patients age 65 and older access over 10 ambulatory visits each year^4^ and geriatrics is a required experience per the Accreditation Council for Graduate Medical Education,^5^ few IM residency programs provide a structured geriatrics curriculum to address these concerns.^6^ For example, in 2006, Warshaw, Bragg, Thomas, Ho, and Brewer revealed that out of 235 IM residency programs, the majority required only 13–24 hours (44%) of dedicated geriatrics education and that there were only 3.5 full-time dedicated geriatric teaching physicians per training program.^6^

In Fall 2017, we performed a needs assessment at the Renaissance School of Medicine at Stony Brook University (response rate: 78%, *N* = 66) and found that a majority of our IM residents similarly had poor knowledge and low confidence in addressing several core women's health conditions. Mean knowledge scores were 56% (*SD* = 24%), and the majority of incorrectly answered questions were about geriatric women's health, such as management of menopause and osteoporosis. Residents were more likely to report feeling unconfident/somewhat unconfident than confident/somewhat confident in 18 of 30 core women's health topics, including management of menopause, abnormal vaginal bleeding, and urinary incontinence (UI). In addition, less than 10% were satisfied with our program's current women's health education, and a majority (78%, *n* = 52) reported that they would be likely/very likely to provide women's health counseling if they received formal training during residency.

Although there is a clear need for geriatric women's health training amongst IM residents, there is limited published curricula for IM residents. Per Zhang, Insetta, Caufield-Noll, and Levine's 2019 review, most women's health curricula (69%) use one time traditional lecture-based didactics, and 38% use case-based small-group sessions.^7^ To our knowledge, there is a paucity of data on geriatric women's health curricula that use interactive, team-based learning, such as the jigsaw teaching method, which is a unique cooperative learning strategy where peers deliver specific content in teams.^8^ In a meta-analysis of cooperative learning methods, the jigsaw teaching method was rated as an easy strategy to learn, initiate, and maintain, as well as being adaptable to changing learning environments.^9^ Learners favor the jigsaw teaching method over traditional didactics because it promotes active learning and enhances their perception of their problem-solving and communication skills.^10^ Current literature has shown this strategy to be successful in pharmacy, dental, nursing, and undergraduate medical education.^10–13^

To date, the jigsaw teaching method has not been used for teaching resident trainees about women's health. In *MedEdPORTAL,* there is only one publication that uses the jigsaw teaching method to teach medical students about pulmonary infections,^14^ and there are no *MedEdPORTAL* publications that educate IM residents about geriatric women's health topics.

Here, we present a 2-hour geriatric women's health jigsaw workshop that aims to increase categorical IM residents’ knowledge and confidence in addressing menopause, osteoporosis, UI, and abnormal uterine bleeding (AUB). We aim to build on the use of the jigsaw teaching method in medical education and to provide a structured geriatric women's health curriculum for IM learners.

## Methods

### Target Audience

We delivered our geriatric women's health workshop to all categorical PGY 1, PGY 2, and PGY 3 IM residents at the Renaissance School of Medicine at Stony Brook University over a 5-week period in January 2018. Our residency program followed a 4+1 block schedule, and these workshops were given as part of its mandatory academic half-day activity. In order to capture all ambulatory groups, we delivered a total of five consecutive 2-hour workshops. This session could be delivered to trainees at various clinical experience levels and in other training programs (i.e., medical students, resident physicians, nurse practitioner students, etc.).

### Venue

This workshop was best delivered in a large group of at least 16 attendees. Participants were divided into small groups of four. Ideally, the classroom had to be large enough for small groups to move to different areas for separate discussions.

### Workshop Implementation Overview

The jigsaw teaching method is a cooperative learning strategy where peers work interdependently in teams and are assigned to develop expertise and then deliver specific content to their peers. In this session, learners were assigned to become content experts in one of four geriatrics women's health topics: (1) menopause, (2) osteoporosis, (3) UI, or (4) AUB. By the end of the session, learners were instructed to collectively apply their knowledge to solve patient cases. The teaching materials included review journal articles on the selected geriatrics women's health topics (Appendix A),^15–22^ student worksheets for each targeted topic (Appendices B–E), patient case worksheets (Appendix F), and facilitator guides for each expert group (Appendices G–J), and patient cases (Appendix K). A typical session ran for 120 minutes.

Please see Appendix L for general facilitator instructions and a schematic of the jigsaw teaching method and Appendix M for an instructional PowerPoint that can be displayed to learners during the session.

### Jigsaw Session Implementation

#### Introduction/home-group assignments

Learners were divided into groups of 4, which were designated as their home groups. These home groups could be preassigned or made at the very beginning of the session. Instructors provided an overview of workshop activities, and learners were instructed to assign each home-group member to be a content expert in one of the four target topics (e.g., learner 1 was assigned to be the menopause expert, learner 2 was the osteoporosis expert, etc.). Instructors informed learners that they would need to use their collective knowledge in their home groups to solve a patient case at the end of the session.

#### Expert-group exercise

Once expert topics were assigned, learners split from their home groups to join members of other home groups who had been given the same assignment topic in order to form expert groups. In the expert group, learners used review journal articles on their selected topic (Appendix A) to complete a guiding worksheet that would facilitate discussion (Appendices B–E). Worksheets included questions regarding a disease topics’ symptoms, diagnostic workup, and management recommendations. Facilitators reviewed worksheet answers and education objectives with each expert group using Appendices G–J.

When choosing articles for each expert topic, the following characteristics were sought: completeness of discussion (i.e., areas addressed included diagnosis, workup, management), ease of reading, concise length such that the article could be read in the time allotted, and applicability to a general internist. We aimed to select one all-inclusive article for each topic, but in some cases, we needed to use more than one review article. The review articles found in the *Annals of Internal Medicine* In the Clinic series and in *American Family Physician* frequently met the characteristics we sought out and were written for primary care physicians. For the UI expert group, there were no articles in either of those sources, and so, we included some subspecialty resources. Given that the subspecialty articles were more detailed than was required for a general internist, we helped learners focus on educational objectives by highlighting applicable article sections and including article page numbers in student worksheets.

#### Patient case activity in home groups

After completing expert worksheets, learners returned to their initial home groups and were instructed to work through two patient cases (Appendix F). Each case highlighted important diagnostic and management points for each learning topic. When reviewing the cases, learners applied the knowledge they had obtained from the expert-group activity to help answer questions about the cases.

#### Summary debriefing

To review the cases and teaching points, all home groups joined together to have a debriefing exercise with faculty facilitators (Appendix K). Our workshop was facilitated by one outpatient general IM physician and one geriatrician. To make the debriefing interactive, we used the Kahoot! education platform,^23^ a free web-based learning program with which educators could create interactive learning games that trainees could play on a smartphone, tablet, or computer. Our Kahoot! learning game included 21 multiple-choice questions about the patient cases, and facilitators allowed learners to defend their answer choices or ask questions to clarify concepts (Appendix K). Using the Kahoot! app is optional. Educators can simply use the debriefing questions to facilitate large-group discussions or might consider using other visual aids, such as Microsoft PowerPoint or Poll Everywhere,^24^ to display questions.

### Workshop Agenda

• Total time: 120 minutes.
• Introduction and home-group assignments: 10 minutes.
• Expert-group activity with individualized tasks: 40 minutes.
  ○ Students complete individual task: 30 minutes.
  ○ Facilitators review answers with each expert group: 10 minutes.
• Home groups review patient cases: 40 minutes.
• Debrief: 30 minutes.

### Personnel

This workshop included all levels of IM resident learners. At least one faculty facilitator was required for the session, but ideally, sessions should have two facilitators. Our workshop was supervised by one general IM physician and one geriatrician. Faculty members could be physicians from IM, family medicine, or obstetrics/gynecology.

### Equipment

The classroom where the workshop takes place should include a computer and a large display, such as a projector with a screen or large-screen monitor. If educators decide to create a Kahoot! quiz, the computer must have access to the internet as the Kahoot! resources are only available online.

Learners needed pen/pencils to complete worksheets and access to a smartphone, tablet, or computer to participate in the Kahoot! game. If there is limited access to electronic equipment, facilitators can ensure that each home group has one team member with a smartphone, tablet, or computer in order to participate in the final Kahoot! exercise.

### Evaluation

To assess the efficacy of this curriculum in improving IM resident knowledge and confidence in managing geriatric women's health conditions, we invited learners to complete a voluntary and anonymous 21-question pretest (Appendix N) immediately before the workshop and a 26-question posttest (Appendix O) immediately after the workshop. The pre- and posttests included questions about demographics and history of women's health training, 12 multiple-choice knowledge questions based on the targeted geriatric women's health topics, and a confidence question about managing women's health based on a 5-point Likert scale. The posttest also asked residents for feedback about the curriculum. Participants were invited to complete the pre- and posttests electronically via Qualtrics or on paper. Questions for each evaluation were created by study investigators and had not been previously validated. We compared baseline and postworkshop responses using the chi-square test and Wilcoxon signed rank test.

## Results

Seventy-four of 84 categorical IM residents participated in our geriatric women's health workshop. All attendees completed the pretest (response rate: 88%), and 62 (74%) completed the posttest. At pretest, there was an even distribution of all PGY levels. The majority of residents were male (*n* = 46, 62%), were of Asian/Pacific Islander descent (*n* = 32, 43%), were from allopathic medical schools (*n* = 60, 82%), and had had previous women's health training during medical school (*n* = 55, 74%; see the Table).

**Table.**
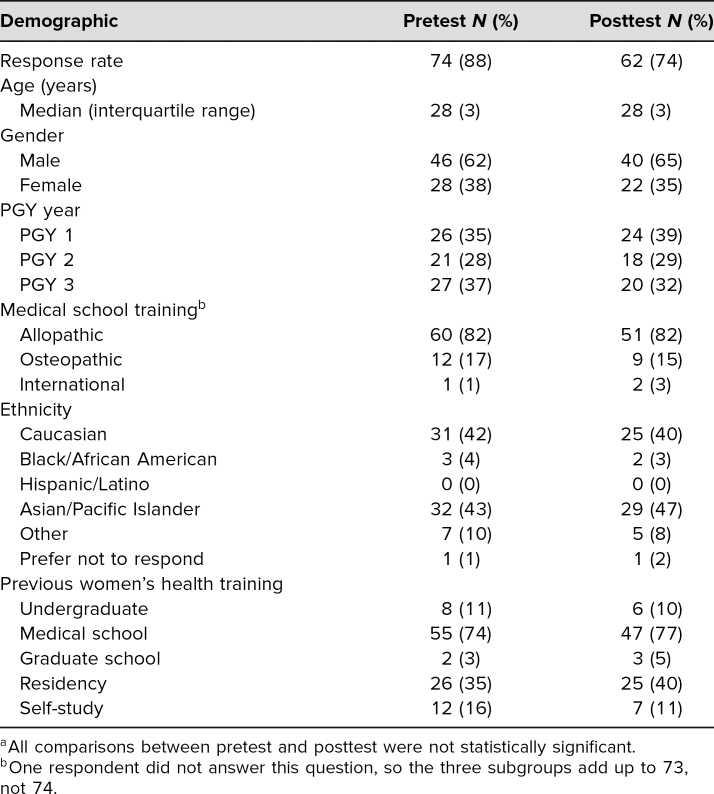
Demographics of Study Population for Pretest and Posttest^a^

**Figure 1.**
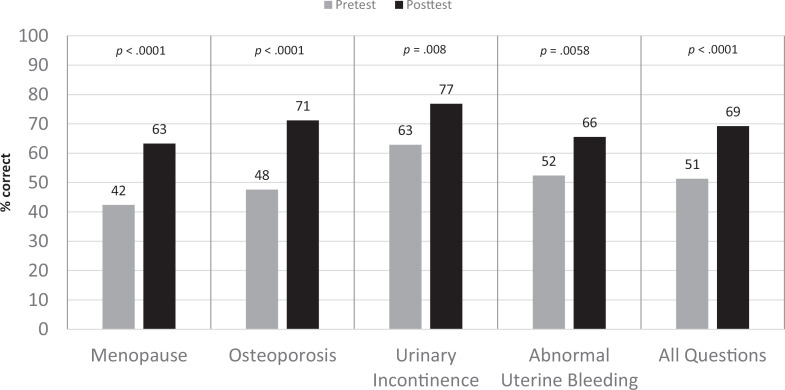
Change in residents’ mean knowledge scores before and after the education workshop. When comparing pretest versus posttest for all questions and each subject, *p* < .05.

**Figure 2.**
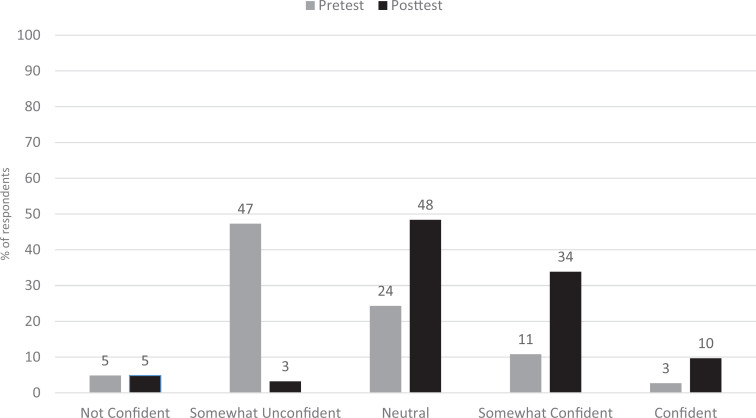
Change in internal medicine residents’ confidence in women's health counseling. *p* < .0001.

At posttest, there was improvement in both knowledge and confidence in addressing geriatric women's health topics. Overall mean knowledge scores increased from 51% to 69% correct (*p* < .0001). When comparing knowledge scores by topic, all scores showed improvement, with the most significant increase in knowledge for osteoporosis (48% to 71%, *p* < .0001) and menopause (42% to 63%, *p* < .0001; see Figure 1). Residents were also more likely to report feeling somewhat confident or confident in counseling a patient on women's health issues (14% vs. 44%, *p* < .0001; see Figure 2).

Furthermore, a majority (94%) of residents reported being satisfied or very satisfied with our geriatric women's health workshop. Qualitative data were overall positive: Residents enjoyed several didactic features such as the topics covered, the interactive team-based structure (jigsaw teaching method), and the Kahoot! summary quiz platform. Residents’ suggestions for improvement included more time to complete expert worksheets and having access to reading materials ahead of time.

## Discussion

To our knowledge, this is the first publication that uses the jigsaw teaching method to educate IM residents about geriatric women's health conditions. Our curriculum was innovative in that we applied a unique cooperative learning strategy with gamification and were able to successfully increase IM residents’ knowledge and confidence in the diagnosis and management of menopause, osteoporosis, UI, and AUB. We were able to achieve our study goals because the jigsaw teaching method required that learners immediately apply knowledge through cases and peer teaching and there was reinforcement of concepts through gamification. Residents who had their continuity clinic at our Veterans Affairs location were the most satisfied with this curriculum because they felt that they had the largest women's health gap prior to the session. Although this workshop was targeted for IM residents, the session could easily be delivered to medical students, residents, fellows or faculty in other primary care specialties such as family medicine, medicine/pediatrics, or obstetrics/gynecology.

Like prior studies, we found the jigsaw teaching method to have several advantages, including that it was easy to learn and implement, as well as being flexible to classroom changes. For example, we preassigned learners to their home groups and expert topics prior to the session, but if there was an unexpected absence or addition, we could readily reorganize teams. We also found that the workshop required only one facilitator if other personnel were not available and that it could cover a wider range of topics. In addition, the learning activities did not depend on learners completing presession assignments, and because each learner was dedicated to a specific topic for a team, every individual had to participate in the session.

By covering multiple topics, we were challenged to ensure that learners achieved all educational objectives within the allotted time. The few critical comments from learners noted that they felt rushed to finish activities and requested to have access to reading before the session. Timing reminders were helpful to keep residents on task, but we suggest that learners preread their articles and that reading materials be prehighlighted to emphasize main teaching points. If educators cannot schedule a full 2-hour workshop, they can require learners to complete worksheets prior to the session and use the expert-group activity to review answers. Educators can alter this session to focus on fewer education topics or shorten the student worksheets with more focused questions. Furthermore, session timing can be shortened if learners complete pre- or posttests at home, and knowledge question stems can be shortened.

Our study had several strengths, including a high response rate, but there were some limitations. Our intervention was applied only to IM residents at a single institution and was not designed to assess whether the jigsaw teaching method was superior to traditional didactics in women's health given the lack of a control group. However, as is often the case in medical education research, it would have been difficult to implement an intervention for only part of a class when the intervention was hypothesized to be beneficial for learner growth and development.

In addition, we did not assess residents’ ability to apply their knowledge in a clinical setting. Future plans to further improve our assessment of this curriculum at a patient care level include creating direct observation tools or standardized patient OSCEs to evaluate trainees in their management of geriatric women's health conditions.

Moving forward, we plan to continue to develop and assess a longitudinal women's health curriculum that will include additional workshops using the jigsaw teaching method. We plan to assess whether the jigsaw teaching method can be effective in increasing knowledge and confidence of IM trainees in other women's health topics. We hope that this curriculum can serve as model for other training programs that include women's health in their core didactics and use academic half-days in their didactic scheduling.

## Appendices

Expert Group Reading Materials.docxStudent Worksheet-Group A AUB.docxStudent Worksheet-Group B Osteoporosis.docxStudent Worksheet-Group C Menopause.docxStudent Worksheet-Group D UI.docxStudent Worksheet-Patient Cases.docxFacilitator Guide-Group A AUB.docxFacilitator Guide-Group B Osteoporosis.docxFacilitator Guide-Group C Menopause.docxFacilitator Guide-Group D UI.docxFacilitator Guide-Patient Cases and Debriefing Questions.docxFacilitator Guide Overview and Jigsaw Instructions.docxGeriatric Women's Health for IM Residents.pptxPretest.docxPosttest.docx All appendices are peer reviewed as integral parts of the Original Publication.
