# Supplementary material for: Using the Jigsaw Teaching Method to Enhance Internal Medicine Residents' Knowledge and Attitudes in Managing Geriatric Women's Health
Source: MedEdPORTAL. 2020 Oct 23;16:11003. doi: 10.15766/mep_2374-8265.11003 (PMC7586752; doi:10.15766/mep_2374-8265.11003)
Supplement: Supplementary file 1 — Expert Group Reading Materials.docxStudent Worksheet-Group A AUB.docxStudent Worksheet-Group B Osteoporosis.docxStudent Worksheet-Group C Menopause.docxStudent Worksheet-Group D UI.docxStudent Worksheet-Patient Cases.docxFacilitator Guide-Group A AUB.docxFacilitator Guide-Group B Osteoporosis.docxFacilitator Guide-Group C Menopause.docxFacilitator Guide-Group D UI.docxFacilitator Guide-Patient Cases and Debriefing Questions.docxFacilitator Guide Overview and Jigsaw Instructions.docxGeriatric Women's Health for IM Residents.pptxPretest.docxPosttest.docx [file mep_2374-8265.11003-s001.zip › A. Expert Group Reading Materials.docx]

**Expert Group Reading Materials**

Below are the list of topic resources for each expert group. Educators should provide copies of these articles to each assigned expert group.

When choosing articles for each expert topic, the following characteristics were sought: completeness of discussion (i.e. addresses areas including diagnosis, work-up, management), ease of reading, concise length such that the article can be read in the time allotted, and applicability to a general internist. We aimed to select one all-inclusive article for each topic, but in some cases we needed to use more than one review article. The review articles found in the Annals of Internal Medicine “In the Clinic” series and in American Family Physician frequently met the characteristics we sought out and both are written for primary care physicians. For the UI expert groups, there were no articles in either of those sources and thus we included some subspecialty resources. Given that the subspecialty articles were more detailed than what is required for a general internist, we helped learners focus on educational objectives by highlighting applicable article sections and included article page numbers in student worksheets.

**Group A: Abnormal Uterine Bleeding**

- Sweet, M et al. Evaluation and Management of Abnormal Uterine Bleeding in Premenopausal Woman. *American Family Physician.* 2012;85(1): 35-42.
- Bradley, L and Gueye, N. The medical management of abnormal uterine bleeding in reproductive-aged women. *American Journal of Obstetrics & Gynecology.* 2016: 31-44.
- Jameson J, Fauci AS, Kasper DL, Hauser SL, Longo DL, Loscalzo J. Figure 385-8 in Disorders of the Female Reproductive System, Harrison's Principles of Internal Medicine, 20e; 2018. Available at: https://accessmedicine.mhmedical.com/ViewLarge.aspx?figid=192287783&gbosContainerID=0&gbosid=0&groupID=0 Accessed: November 03, 2019

**Group B: Osteoporosis**

- Ensrud, K and Crandall, C. Osteoporosis. *Ann Intern Med*. 2017;167:ITC17–ITC32.
- Black, D. and Rosen, C. Postmenopausal Osteoporosis. *N Engl J Med* 2016;374:254-62.

**Group C: Menopause**

- Col, N. et al. In The Clinic: Menopause. *Ann Intern Med*. 2009;ITC4-16.

**Group D: Urinary Incontinence**

- Hersh, L and Salzman, B. Clinical Management of Urinary Incontinence in Women. *American Family Physician*. 2013; 87(9): 635-640.
- Menefee, S. and Nygaard, I. 2012. “Chapter 26 Lower Urinary Tract Disorders.” Ed. J. Berek and D. Berek. Philadelphia: Lippincott Williams & Wilkins, 2012. 862-874, 884-888.
