# Supplementary material for: Using the Jigsaw Teaching Method to Enhance Internal Medicine Residents' Knowledge and Attitudes in Managing Geriatric Women's Health
Source: MedEdPORTAL. 2020 Oct 23;16:11003. doi: 10.15766/mep_2374-8265.11003 (PMC7586752; doi:10.15766/mep_2374-8265.11003)
Supplement: Supplementary file 1 — Expert Group Reading Materials.docxStudent Worksheet-Group A AUB.docxStudent Worksheet-Group B Osteoporosis.docxStudent Worksheet-Group C Menopause.docxStudent Worksheet-Group D UI.docxStudent Worksheet-Patient Cases.docxFacilitator Guide-Group A AUB.docxFacilitator Guide-Group B Osteoporosis.docxFacilitator Guide-Group C Menopause.docxFacilitator Guide-Group D UI.docxFacilitator Guide-Patient Cases and Debriefing Questions.docxFacilitator Guide Overview and Jigsaw Instructions.docxGeriatric Women's Health for IM Residents.pptxPretest.docxPosttest.docx [file mep_2374-8265.11003-s001.zip › B. Student Worksheet-Group A AUB.docx]

**Learning Objectives**:

- Describe the pathophysiology of normal menstruation
- Define anovulatory and ovulatory abnormal uterine bleeding (AUB)
- List potential etiologies for abnormal uterine bleeding using the International Federation of Gynecology and Obstetrics (FIGO) classification system
- Describe the risk factors for endometrial carcinoma
- Describe history and physical exam feature that should be obtained when evaluating a patient with AUB
- List the diagnostic tests to order when evaluation a patient with AUB
- Describe the medical and surgical treatment options of AUB

1. **What is the pathophysiology of a normal menstrual cycle? Please include the duration, frequency, and volume of normal menstruation? (Can use the Menstrual Cycle Figure from Harrison’s Principle of Internal Medicine-** **Sweet et al, page 35)**

| *Phase* | *Day* | *Estradiol* | *Progesterone* | *LH* | *FSH* | *Follicle/ovary* | *Endometrium* |
| --- | --- | --- | --- | --- | --- | --- | --- |
| **Follicular** |  |  |  |  |  |  |  |
| Early |  |  |  |  |  |  |  |
| Mid |  |  |  |  |  |  |  |
| Late |  |  |  |  |  |  |  |
| **Luteal** |  |  |  |  |  |  |  |
| Ovulation |  |  |  |  |  |  |  |
| Mid |  |  |  |  |  |  |  |
| Late |  |  |  |  |  |  |  |

1. **What is the definition of abnormal uterine bleeding? (Sweet page 36)**
2. **What is the difference between anovulatory and ovulatory AUB? What are the potential etiologies for each and what age groups often have which type of bleeding****? (Sweet pg 36; Bradley pg 35)**
3. **What are risk factors for endometrial carcinoma? (Sweet pg 36; Bradley pg 24)**
4. **Per the FIGO classification system, what mnemonic can you use to remember the differential diagnosis of AUB?** **(Bradley pg 35)**
5. **What history and physical should you obtain when evaluating a patient for AUB? (Bradley- table 2)**
6. **What diagnostic tests would you order to begin your work up of AUB? (Bradley table 2; Sweet table 1, figure 1 and 2)**
7. **What medications are used to treat AUB? Complete the table below to describe if the medication is used for for anovulatory or ovulatory AUB, if it provides contraception, dosage/formulation, contraindications and side effects. (Use “A” for anovulatory and “O” for ovulatory)- (Bradley Table 3; Sweet Table 3)**

| **Medication** | **AUB type** | **Formulation** | **Contraindications** | **Side Effects** | **Provides contraception** |
| --- | --- | --- | --- | --- | --- |
| ***Hormonal*** | | | | | |
|  |  |  |  |  |  |
|  |  |  |  |  |  |
|  |  |  |  |  |  |
|  |  |  |  |  |  |
|  |  |  |  |  |  |
|  |  |  |  |  |  |
|  |  |  |  |  |  |
| ***Non-Hormonal*** | | | | | |
|  |  |  |  |  |  |
|  |  |  |  |  |  |

1. **What are surgery procedures can treat AUB? (Sweet pg 41)**
