# Supplementary material for: Using the Jigsaw Teaching Method to Enhance Internal Medicine Residents' Knowledge and Attitudes in Managing Geriatric Women's Health
Source: MedEdPORTAL. 2020 Oct 23;16:11003. doi: 10.15766/mep_2374-8265.11003 (PMC7586752; doi:10.15766/mep_2374-8265.11003)
Supplement: Supplementary file 1 — Expert Group Reading Materials.docxStudent Worksheet-Group A AUB.docxStudent Worksheet-Group B Osteoporosis.docxStudent Worksheet-Group C Menopause.docxStudent Worksheet-Group D UI.docxStudent Worksheet-Patient Cases.docxFacilitator Guide-Group A AUB.docxFacilitator Guide-Group B Osteoporosis.docxFacilitator Guide-Group C Menopause.docxFacilitator Guide-Group D UI.docxFacilitator Guide-Patient Cases and Debriefing Questions.docxFacilitator Guide Overview and Jigsaw Instructions.docxGeriatric Women's Health for IM Residents.pptxPretest.docxPosttest.docx [file mep_2374-8265.11003-s001.zip › C. Student Worksheet-Group B Osteoporosis.docx]

**Learning Objectives**:

- Describe the pathophysiology of osteoporosis
- List risk factors for osteoporosis
- Describe screening guidelines for osteoporosis
- Interpret bone mineral density test results and diagnose osteoporosis
- List laboratory tests to order when evaluating for secondary causes of osteoporosis
- Describe lifestyle modifications to prevent and treat osteoporosis
- Describe pharmacologic treatment options for osteoporosis and their potential side effects
- Identify which patients require drug holiday from bisphosphonates

1. **What is osteoporosis? (Annals ITC8)**

1. **What are the USPFTF guidelines for osteoporosis screening? (Annals ITC18)**

1. **When would you consider to screen younger women for osteoporosis? (Annals ITC18)**

1. **How frequently should we screen for osteoporosis? (Annals ITC19)**

1. **List at least 5 risk factors for osteoporosis. (Annals ITC19- Box)**

1. **What is the diagnostic criteria for osteoporosis? (Annals ITC22)**

1. **What’s the difference between the T-score and Z-score? When would you use each? (Annals ITC22)**

1. **What are secondary causes of osteoporosis and what tests can you order to work these up? (Annals ITC23)**
2. **What lifestyle recommendations can you provide patients to prevent osteoporosis? (Annals ITC21)**

1. **What is the recommended daily intake of vitamin D and calcium for women? (Annals ITC21)**

1. **Complete the table below to describe the pharmacologic treatments available for osteoporosis. (Can describe medications as family classes; NEJM- pg 257)**

| **Medication** | **Who should receive** | **Administration** | **Mechanism of Action** | **Side Effects/ Contraindications** |
| --- | --- | --- | --- | --- |
|  |  |  |  |  |
|  |  |  |  |  |
|  |  |  |  |  |
|  |  |  |  |  |
|  |  |  |  |  |
|  |  |  |  |  |

1. **What is the risk of developing osteonecrosis of the jaw from bisphosphonates and what factors increase a patient’s risk? (Annals ITC28)**

1. **When should patients receive a drug holiday from bisphosphonates? (Annals ITC28)**
