# Supplementary material for: Using the Jigsaw Teaching Method to Enhance Internal Medicine Residents' Knowledge and Attitudes in Managing Geriatric Women's Health
Source: MedEdPORTAL. 2020 Oct 23;16:11003. doi: 10.15766/mep_2374-8265.11003 (PMC7586752; doi:10.15766/mep_2374-8265.11003)
Supplement: Supplementary file 1 — Expert Group Reading Materials.docxStudent Worksheet-Group A AUB.docxStudent Worksheet-Group B Osteoporosis.docxStudent Worksheet-Group C Menopause.docxStudent Worksheet-Group D UI.docxStudent Worksheet-Patient Cases.docxFacilitator Guide-Group A AUB.docxFacilitator Guide-Group B Osteoporosis.docxFacilitator Guide-Group C Menopause.docxFacilitator Guide-Group D UI.docxFacilitator Guide-Patient Cases and Debriefing Questions.docxFacilitator Guide Overview and Jigsaw Instructions.docxGeriatric Women's Health for IM Residents.pptxPretest.docxPosttest.docx [file mep_2374-8265.11003-s001.zip › D. Student Worksheet-Group C Menopause.docx]

**Learning Objectives**

- Define menopause and when it typically occurs in women
- Identify factors that affect the onset of menopause
- List the stages of menopause
- Describe symptoms associated with menopause and how the diagnosis of menopause is made
- Describe lifestyle modifications to treat the common menopause symptoms
- Describe the indications, side effects, and contraindications of hormone replacement therapy
- List and describe non-hormonal medication options for managing menopause
- Describe the complementary-alternative medicine options for menopause symptom relief

1. **What is menopause? (ITC 4-2)**

1. **When does menopause occur? (ITC 4-2)**

1. **What factors affect age of onset of menopause? (ITC 4-2)**

1. **What are the stages of menopause? (ITC 4-2, Table 1)**

1. **What symptoms suggest menopause and over what time period do they occur? (ITC 4-2, ITC 4-3)**

1. **What are the diagnostic criteria for menopause? (ITC 4-3)**

1. **What are lifestyle modifications that can relieve menopausal symptoms? (ITC 4-4)**
2. **What hormonal medications can treat menopause? What is the dosing and duration of therapy? (ITC 4-9)**
3. **What are side effects of HRT and what should be monitored when a patient is on HRT? (ITC 4-7 to ITC 4-8)**

1. **What non-hormonal treatments are available for menopause? (ITC 4-10, Table 6)**
