# Supplementary material for: Using the Jigsaw Teaching Method to Enhance Internal Medicine Residents' Knowledge and Attitudes in Managing Geriatric Women's Health
Source: MedEdPORTAL. 2020 Oct 23;16:11003. doi: 10.15766/mep_2374-8265.11003 (PMC7586752; doi:10.15766/mep_2374-8265.11003)
Supplement: Supplementary file 1 — Expert Group Reading Materials.docxStudent Worksheet-Group A AUB.docxStudent Worksheet-Group B Osteoporosis.docxStudent Worksheet-Group C Menopause.docxStudent Worksheet-Group D UI.docxStudent Worksheet-Patient Cases.docxFacilitator Guide-Group A AUB.docxFacilitator Guide-Group B Osteoporosis.docxFacilitator Guide-Group C Menopause.docxFacilitator Guide-Group D UI.docxFacilitator Guide-Patient Cases and Debriefing Questions.docxFacilitator Guide Overview and Jigsaw Instructions.docxGeriatric Women's Health for IM Residents.pptxPretest.docxPosttest.docx [file mep_2374-8265.11003-s001.zip › E. Student Worksheet-Group D UI.docx]

**Learning Objectives**

- Define urinary incontinence (UI)
- Describe the prevalence and costs of UI
- Describe the effects that UI has on an individual’s life
- Describe how micturition is controlled and list the different types of incontinence that can develop
- List risk factors for developing incontinence
- Describe the evaluation and work-up of incontinence
- Describe nonsurgical treatments available for incontinence including those recommended lifestyle modifications
- List pharmacologic options for incontinence and describe the adverse effects associated with these treatments

1. **What is Urinary Incontinence? (AFP Page 634)**

1. **How prevalent is UI and how does it affect the patient and society? (AFP page 634)**

1. **What factors control micturition? (Berek Page 862)**

1. **What types of urinary incontinence are there? (AFP page 635, Table 1)**

1. **What are the risk factors for incontinence? (Berek Page 871)**

1. **What is the work-up and evaluation for urinary incontinence? (Berek Page 871-877)**

1. **What nonsurgical treatment is available for urinary incontinence? (AFP page 635: table 2, AFP page 636: table 3)**

1. **What medications are used for the treatment of urinary incontinence?** (**Berek Page 888 table 26.9, AFP page 635: table 2)**

1. **What are the side effects of the medications for urge incontinence? (AFP Page 637)**
