# Supplementary material for: Using the Jigsaw Teaching Method to Enhance Internal Medicine Residents' Knowledge and Attitudes in Managing Geriatric Women's Health
Source: MedEdPORTAL. 2020 Oct 23;16:11003. doi: 10.15766/mep_2374-8265.11003 (PMC7586752; doi:10.15766/mep_2374-8265.11003)
Supplement: Supplementary file 1 — Expert Group Reading Materials.docxStudent Worksheet-Group A AUB.docxStudent Worksheet-Group B Osteoporosis.docxStudent Worksheet-Group C Menopause.docxStudent Worksheet-Group D UI.docxStudent Worksheet-Patient Cases.docxFacilitator Guide-Group A AUB.docxFacilitator Guide-Group B Osteoporosis.docxFacilitator Guide-Group C Menopause.docxFacilitator Guide-Group D UI.docxFacilitator Guide-Patient Cases and Debriefing Questions.docxFacilitator Guide Overview and Jigsaw Instructions.docxGeriatric Women's Health for IM Residents.pptxPretest.docxPosttest.docx [file mep_2374-8265.11003-s001.zip › F. Student Worksheet-Patient Cases.docx]

**Case 1**- Mrs. Johnson is a 48 year old with history of diabetes, hypertension, and allergic rhinitis who presents with difficulty sleeping. Her symptoms started a few months ago where she would have trouble staying asleep all night due to episodes of feeling extremely hot and sweaty. She has tried layering clothes, opening windows and using fans in her room but nothing seems to help. Her sleep is also disturbed by her constant urge to go to the bathroom. This increased urgency also occurs during the day and feels that she runs to the bathroom every hour. She endorses some vaginal dryness but denies any fever, leaking of urine with coughing, pain with urination, or blood in her urine.

Past Medical History: type 2 diabetes, hypertension, allergic rhinitis

Past Surgical History: None

Gynecologic history:

- LMP: 3 months ago, has been every few months over the past year
- 3 pregnancies. All vaginal deliveries
- Sexual history- 1 sexual partner, admits to some pain with intercourse
- Last pap smear- 2 years ago, no history of abnormal
- Mammogram- 1 year ago, no history of abnormal

Medications: Metformin, Lisinopril, cetirizine

Family History: Mom- diabetes; Dad- coronary artery disease; no family history of cancer or blood clots

Social History: Smokes 5 cigarettes per day. Drinks a glass of wine 3-4 times a week with dinner

Physical Exam is only notable for BMI 35 and vaginal atrophy

In office urinalysis: negative

**1. What are the likely causes for this patient’s symptoms?**

**2. What would you recommend to her to provide relief? If you are prescribing medication, what side effects would you educate her on?**

**Case 2**- Mrs. Bailey is a 67 year old widowed female with past medical history COPD with frequent exacerbations, hyperlipidemia, epilepsy, and past tobacco use who presents to review her recent labs and bone density test. She overall feels well but upon review of symptoms, she mentions intermittent vaginal spotting over the past month. It has occurred at least once weekly and persists for 2-3 days. She denies any vaginal pain, recent intercourse, weight loss, abdominal pain, dysuria, hematuria, or bloody bowel movement. She has never had any issues bleeding in the past, including never having prolong bloody nose, gum bleeding or joint bleeding.

Past Medical History: COPD, epilepsy, HLD, wrist fracture 2 years ago. She has been in the hospital 4 times this past year for COPD exacerbations.

Past Surgical History: Appendectomy- no complications

Gynecologic history:

- Menopause since age 52
- No history of pregnancy
- Sexual history- no current partners. Husband passed away last year and they were married for 35 years
- Last pap smear- stopped after age 65, no history of abnormal paps

Medications: fluticasone/salmeterol inhaler, tiotropium inhaler, phenytoin, pravastatin

Family History: Mom- breast cancer, osteopenia; Dad- hypertension, emphysema

Social history: Retired waitress. Spends most of the day sitting. Used to smoke 1 pack per day for 40 years. Quit last year after she was hospitalized. Social drinkers- 1-2 drinks every few weeks

Physical Exam notable for:

- BMI 18.3, Ht 5’5”, Wt 105lbs, BP 100/70, HR 90
- HEENT- Pale conjunctiva
- Lungs- Distant lung sounds, prolong expiration
- Bimanual exam- unremarkable, no blood in vaginal vault
- Remainder exam normal

Labs:

- CBC- WBC 8.2, Hg 9.5, Hct 29.2, Plt 224
- BMP- Na 135, K 4, Cl 105, CO2 25, BUN 22, Cr 1.0
- LFT- AST 23, ALT 26, AP 88, TP 7.2, TB 1.1
- TSH 0.7
- INR 1.3
- Vitamin D level 32

DEXA Results:

| **Location** | **T-Score** | **Z-score** | 10-year risk of major osteoporotic fracture = 16%  10-year risk of hip fracture = 3.9% |
| --- | --- | --- | --- |
| Trochanter | -2.0 | -2.5 |  |
| Femoral neck | -1.9 | -2.3 |  |
| L1-L4 | -0.77 | -0.57 |  |

1. **What risk factors does this patient have for osteoporosis?**
2. **What is your interpretation of her bone density test? What would you recommend to this patient regarding her bone health? If you are prescribing a medication, how long will she need to be on the medication and what side effects would you educate her about?**
3. **What is your assessment of her vaginal bleeding? What would be your next steps?**
4. **If her endometrial biopsy returned as hyperplasia without atypia, what would her treatment options include?**
