# Supplementary material for: Using the Jigsaw Teaching Method to Enhance Internal Medicine Residents' Knowledge and Attitudes in Managing Geriatric Women's Health
Source: MedEdPORTAL. 2020 Oct 23;16:11003. doi: 10.15766/mep_2374-8265.11003 (PMC7586752; doi:10.15766/mep_2374-8265.11003)
Supplement: Supplementary file 1 — Expert Group Reading Materials.docxStudent Worksheet-Group A AUB.docxStudent Worksheet-Group B Osteoporosis.docxStudent Worksheet-Group C Menopause.docxStudent Worksheet-Group D UI.docxStudent Worksheet-Patient Cases.docxFacilitator Guide-Group A AUB.docxFacilitator Guide-Group B Osteoporosis.docxFacilitator Guide-Group C Menopause.docxFacilitator Guide-Group D UI.docxFacilitator Guide-Patient Cases and Debriefing Questions.docxFacilitator Guide Overview and Jigsaw Instructions.docxGeriatric Women's Health for IM Residents.pptxPretest.docxPosttest.docx [file mep_2374-8265.11003-s001.zip › G. Facilitator Guide-Group A AUB.docx]

***Note to Facilitators:*** *During the expert group activity, please circulate the room to ensure that learners are discussing accurate teaching points. You should provide immediate feedback if discussions are off topic or information is incorrect. This document will include take home points for each question, but it is not meant to provide to learners verbatim. During expert review, learners should provide answers to you first and then you can fill in gaps. We included detailed answers in case you are not as familiar with this topic and have one comprehensive reference for the topic.*

**Learning Objectives**:

- Describe the pathophysiology of normal menstruation
- Define anovulatory and ovulatory abnormal uterine bleeding (AUB)
- List potential etiologies for abnormal uterine bleeding using the International Federation of Gynecology and Obstetrics (FIGO) classification system
- Describe the risk factors for endometrial carcinoma
- Describe history and physical exam feature that should be obtained when evaluating a patient with AUB
- List the diagnostic tests to order when evaluation a patient with AUB
- Describe the medical and surgical treatment options of AUB

**1) What is the pathophysiology of a normal menstrual cycle? Please include the duration, frequency, and volume of normal menstruation? (Can use image from Harrison’s for reference; Sweet et al, page 35)**

Normal menstruation:

- Duration: 4 – 7 days
- Frequency: Every 24-38 days
- Volume: Mean 35 cc blood loss. Total volume is about 70cc

| *Phase* | *Day* | *Estradiol* | *Progesterone* | *LH* | *FSH* | *Follicle/ovary* | *Endometrium* |
| --- | --- | --- | --- | --- | --- | --- | --- |
| **Follicular** | 0-14 |  |  |  |  |  |  |
| Early | 0-7 | Low | Low | Low | Low, slow increase | Corpus abicans- dies off | Menses |
| Mid | 9-10 | Increasing | Low | Low | Increasing | FSH stimulates dominate follicle, which releases estradiol | Start proliferation of endometrium |
| Late | 10-14 | Increasing | Low | Rising | Slowly rising | Dominant follicle continues to secrete estradiol | Proliferation of endometrium |
| **Luteal** | 14-28 |  |  |  |  |  |  |
| Ovulation | 14 | High/peak | Increasing | High | High/peak | Follicle rupture, oocyte released. Corpus luteum releases progesterone | Stop mitosis and organize glands |
| Mid | 15-21 | Decreasing | Increasing | Decrease | Decrease | Corpus luteum releases progesterone | Endometrium continues to organize |
| Late | 22-28 | Low | Low | Low | Low | If no fertilization, estradiol and progesterone will not be produced | Blood supply lost |

*Talking points- Learners should have a general overview of a normal menstrual cycle, when hormones increase/decrease and what changes occur to the follicles and endometrium. Having this background will allow them to understand what is an abnormal cycle / abnormal uterine bleeding.*

**2) What is the definition of abnormal uterine bleeding? (Sweet pg 36)**

AUB is uterine bleeding that occurs different from normal menstrual flow in regard to volume, frequency, and duration in the absence of pregnancy. It can be described as anovulatory or ovulatory AUB.

*Talking Points:* *AUB is any aberration from normal menstrual flow. The above image is a nice review but not required for learners to know/see*

**3) What is the difference between anovulatory and ovulatory AUB? What are the potential etiologies for each and what age groups often have which type of bleeding? (Sweet pg 36/40; Bradley pg 35)**

Anovulatory AUB: Menses occur irregularly or infrequently and may be light or heavy flow.

- *Pathophysiology*: Related to dysfunction of the normal rise and fall of estrogen and progesterone. Usually there is estrogen-primed endometrial proliferation but no progesterone to help with stabilize the endometrium, thereby leading to unpredictable periods
- *Age*: Adolescence at the start of menarche and in late 40s-50s when patients are perimenopause
- *DDx*:
  - Adolescence
  - Eating disorder
  - Uncontrolled diabetes mellitus
  - PCOS
  - Hyper- or hypothyroidism
  - Hyperprolactinemia
  - Medication effects: Antiepileptics, Antipsychotics
  - Pregnancy
  - Perimenopause
  - Malignancy- endometrial carcinoma
- This group has higher chance of endometrial carcinoma

Ovulatory AUB : Periods occur regular frequencies but menses is usually very heavy or last longer than 7 days (ex. Menorrhagia).

- *Pathophysiology:* There is often overproduction of prostaglandins and dysregulation for vasoconstriction of the endometrial lining.
- *Age*: More common in younger women
- *DDx*
  - Bleeding disorder: Factor deficiency, Leukemia, Platelet disorder, von Willebrand disease
  - Hypothyroidism
  - Liver disease, advanced
  - Structural lesions:
    - Benign-Fibroids, Polyp, Adenomyosis, hyperplasia
    - Malignancy- endometrial carcinoma (less common in this group)

*Talking points: Anovulatory AUB is mostly irregular, in older women, and associated with cancer or endocrinopathies. Ovulatory AUB involves regular heavy periods, structural abnormalities and coagulopathies.*

**4) What are risk factors for endometrial carcinoma?** **(Sweet pg 36; Bradley pg 24)**

- Recurrent anovulation
- Advanced age (>50 years old)
- Obesity
- Nulliparity
- Infertility
- Diabetes
- Family history of breast, endometrial, colon cancer
- Long-term unopposed estrogen therapy
- History of tamoxifen use

*Talking Points: Should emphasizes advanced age and unopposed estrogen*

**5) Per the FIGO classification system, what mnemonic can you use to remember the differential diagnosis of AUB? (Bradley pg 35)**

PALM-COEIN

- PALM: Polyps, Adenomyosis, Leimyoma, Malignancy
- COEIN: Coagulopathy, Ovulatory disorders, Endometrial, Iatrogenic, Not Classified
- Refer to Figure in Bradley, L and Gueye, N. (2016). The medical management of abnormal uterine bleeding in reproductive-aged women. *American Journal of Obstetrics & Gynecology.* 2016: 31-44.

*Talking Points: see above*

**6) What history and physical should you obtain when evaluating a patient for AUB?**

Please refer to Table 2: Focused assessment of abnormal uterine bleeding in Bradley, L and Gueye, N. (2016). The medical management of abnormal uterine bleeding in reproductive-aged women. *American Journal of Obstetrics & Gynecology.* 2016: 31-44.

Screening questions for coagulation disorders (positive screen if 1 is true)

- Heavy periods since menarche
- 1 of the following
  - Postpartum hemorrhage
  - Surgery-related bleeding
  - Bleeding associated with dental work
- 2 or more of the following
  - Bruising 1-2x/month
  - Epistaxis 1-2x/month
  - Frequent gum bleeding
  - FHx of bleeding sx

If pt is post-menopausal, should ask if pt is on HRT since being on HRT can cause spontaneous bleeding. Medication may only need adjustment.

*Talking Points: Emphasize importance of reviewing medication history and considering malignancy in post-menopausal females.*

**7) What diagnostic tests would you order to begin your work up of AUB? (Bradley table 2; Sweet table 1, figure 1 and 2)**

Initial Labs:

- CBC
- B-HCG serum or urine (need to confirm patient is not pregnant)
- Pap smear- especially if female c/o post-coital bleeding
- GC/Chlamydia- vaginal culture

Additional tests as clinically indicated:

- TSH
- Iron studies- if anemic
- Prolactin- especially if considering hyperprolactinemia or antipsychotics
- LFT
- PT/PTT/INR
- Fibrinogen
- vWD w/u: vWF antigen, Ristocetin cofactor, Factor VIII (VWD present in up to 11% of women)

If concerned for PCOS, check for serum androgens

- Total testosterone (>60)
- AM serum 17-hydroxyprogesterone
- FSH
- LH
- Estradiol

Endometrial biopsy

- Post-menopausal
- If pt is 45 yo and older
- <45 yo and has unopposed estrogen stimulation or has failed medical management

Imaging

- Transvaginal US
  - Check for structural uterine abnormalities such as PCOS, fibroids, polyps, endometrial thickening, etc.
  - If endometrial lining >5mm, pt should be referred for endometrial biopsy or D+C sample
- Hysteroscopy- when referred to GYN

*Talking Points: Emphasize initial labs and need for endometrial biopsy if pt is older than 45 years old or post-menopausal.*

**8) What medications are used to treat AUB? Complete the table below to describe if the medication is used for anovulatory or ovulatory AUB, dosage/formulation, contraindications and side effects. (Use “A” for anovulatory and “O” for ovulatory) (Bradley Table 3; Sweet Table 3)**

First it is essential to correctly diagnose the cause of AUB. The ultimate goals of treatment are to regulate menstrual cycles, minimize blood loss, and improve quality of life.

If cause of bleeding related to endocrine d/o (ex. Hypothyroidism, hyperprolactinemia, PCOS) or coagulopathy, it is important to treat these underlying causes. If AUB related to endometrial carcinoma, pt will need referral to gyn-onc and may need chemotherapy pending how advance the disease is.

| **Medication** | **AUB type** | **Formulation** | **Contraindications** | **Side Effects** | **Provides contraception** |
| --- | --- | --- | --- | --- | --- |
| ***Hormonal*** *(Should consider if pt wants/needs contraception)* | | | | | |
| **Combined estrogen/progesterone contraception**  -Estrogen inhibits FSH, development of dominant follicle, and stabilizes endometrium  -Progestin inhibits LH surge, ovulation and creates atrophic endometrial lining | A, O | -Cyclic monophasic or triphasic OCP  -Extended or continuous monophasic pill  -Vaginal ring (Ex. Nuvaring)  -Transdermal (Ex. OrthoEvra)- 1 patch per week x 3 weeks, 1 patch free week | Pregnancy; smoker; hx of malabsorptive bariatric surgery; hx of VTE, thrombogenic mutations; cardiac risk factors; migraine with aura; prolong immobilization; current or past CAD, CVA, liver disease/tumor, breast CA, | Spotting, nausea, HA, breast tenderness, breakthrough bleeding, HTN, VTE, stroke, MI | Yes |
| **Oral progestin**  -Progesterone stabilizes endometrium, prevents ovulation, and leads to atrophic endometirum | A, O | Ex. medroxyprogeserone acetate (2.5-10 mg),  norethindrone (2.5-5 mg),  megestrol acetate (40-  320 mg), or micronized  progesterone (200-400 mg)  Schedule depends on if anovulatory or ovulatory bleeding  *Anovulatory*: 1 tablet daily for 2 weeks every 4 weeks  *Ovulatory*:  -Cyclic: 1 tab For 21 days per month  -Continuous: 1 tab daily  For women who cannot take estrogen | Pregnant, history of malabsorptive  bariatric surgery, liver disease/tumor, breast CA, ischemic heart disease | Irregular bleeding, increased risk of thrombotic event | No |
| **Depot medroxyprogesterone acetate (DMPA)** | O | DMPA 150mg IM q 12 weeks  **For women who cannot take estrogen | Pregnant, cardiac risk factors, liver disease/tumor, breast CA, ischemic heart disease | Irregular bleeding, amenorrhea, decrease bone density, boating, breast tenderness, fluid retention, increased risk of thrombotic event | Yes |
| **Levonorgesterol IUD** | A, O | Mirena, Liletta, Skyla- lasts 3-6 yrs  Releases 20 ug/day  **Preferred for pts with VTE or on anticoagulation | Pregnant, untreated cervical or uterine CA, large or distorted uterine cavity, PID or STI within 3mo, liver disease or tumor | Irregular bleeding/spotting, cramping, breast tenderness, decrease libido, acne, nausea, increased risk of thrombotic event | Yes |
| **Megestrol** | A | Megace 40mg daily | Liver disease | Prolong use can cause adrenal insufficiency, VTE | No |
| **Leuprolide acetate**  -GnRH agonist that inhibits LH and FSH | O | Lupron 3.75mg IM q monthly  Lupron 11.25mg IM q 3 months | Pregnant | Menopause sx- hot flashes, sweating, vaginal dryness, bone loss (if used >6mo) | No |
| **Danazol**  -Synthetic ethisterone that inhibits FSH and LH, causing thinning of endometrium | O | 100-400mg PO daily (divided doses) | Pregnant, unexplained vaginal  bleeding, impaired hepatic, renal, or  cardiac function | Weight gain, acne, androgenic effects | No |
| ***Non-Hormonal*** | | | | | |
| **NSAIDs**  -Suppress COX and decrease prostaglandin synthesis | O | Ibuprofen- 600-1200mg/day  Naproxen- 500-1100mg/day  Helpful for dysmenorrhea (painful periods) | Pregnant, anaphylaxis, hx of GIB, IBD, CKD, CHF | GI side effects, bleeding | No |
| **Tranexamic acid**  -Blocks plasminogen binding sites, preventing fibrin and clot break down | O | Lysteda 650 mg; two tablets  three times per day,  five days per month  Begin of first day of menses  **Used for pts with hemophilia | Hx of VTE, CKD, active intravascular clotting or ICH, impaired color vision, on OCP | HA, nausea, vomiting, diarrhea, muscle pain, dysmenorrhea | No |

Note: A = anvolulatory; O = ovulatory

*Talking points: Review that there are groups of medication (hormonal and non-hormonal) and that not everyone one can be used for anovulatory and ovulatory AUB. Clinicians should consider if the patient would like contraception and thus choose options that will also provide this feature. Should also emphasize talking points in table above.*

**9) What are surgical options for AUB? (Sweet pg 41)**

- Hysterectomy- definitive treatment for heavy bleeding and choice if does not respond to medical therapy
- Uterine artery embolization- specifically for fibroids
- Polypectomy
- Fibroidectomy/Myomectomy
- Endometrial ablation

See Figures 1 and 2 in Sweet et al. (2012). Evaluation and Management of Abnormal Uterine Bleeding in Premenopausal Woman. American Family Physician 85(1): 35-42, for algorithms in evaluation and treatment of anovulatory uterine bleeding

*Talking points: Remove options above and refer learners to algorithms in Sweet article.*

**References**

- Sweet, M et al. Evaluation and Management of Abnormal Uterine Bleeding in Premenopausal Woman. *American Family Physician.* 2012;85(1): 35-42.
- Bradley, L and Gueye, N. (2016). The medical management of abnormal uterine bleeding in reproductive-aged women. *American Journal of Obstetrics & Gynecology.* 2016: 31-44.
- Jameson J, Fauci AS, Kasper DL, Hauser SL, Longo DL, Loscalzo J. Disorders of the Female Reproductive System, Harrison's Principles of Internal Medicine, 20e; 2018. Available at: https://accessmedicine.mhmedical.com/ViewLarge.aspx?figid=192287783&gbosContainerID=0&gbosid=0&groupID=0 Accessed: November 03, 2019
