# Supplementary material for: Using the Jigsaw Teaching Method to Enhance Internal Medicine Residents' Knowledge and Attitudes in Managing Geriatric Women's Health
Source: MedEdPORTAL. 2020 Oct 23;16:11003. doi: 10.15766/mep_2374-8265.11003 (PMC7586752; doi:10.15766/mep_2374-8265.11003)
Supplement: Supplementary file 1 — Expert Group Reading Materials.docxStudent Worksheet-Group A AUB.docxStudent Worksheet-Group B Osteoporosis.docxStudent Worksheet-Group C Menopause.docxStudent Worksheet-Group D UI.docxStudent Worksheet-Patient Cases.docxFacilitator Guide-Group A AUB.docxFacilitator Guide-Group B Osteoporosis.docxFacilitator Guide-Group C Menopause.docxFacilitator Guide-Group D UI.docxFacilitator Guide-Patient Cases and Debriefing Questions.docxFacilitator Guide Overview and Jigsaw Instructions.docxGeriatric Women's Health for IM Residents.pptxPretest.docxPosttest.docx [file mep_2374-8265.11003-s001.zip › H. Facilitator Guide-Group B Osteoporosis.docx]

***Note to Facilitators:*** *During the expert group activity, please circulate the room to ensure that learners are discussing accurate teaching points. You should provide immediate feedback if discussions are off topic or information is incorrect. This document will include take home points for each question but it is not meant to provide to learners verbatim. During expert review, learners should provide answers to you first and then you can fill in gaps. We included detailed answers in case you are not as familiar with this topic and have one comprehensive reference for the topic.*

**Learning Objectives**:

- Describe the pathophysiology of osteoporosis
- List risk factors for osteoporosis
- Describe screening guidelines for osteoporosis
- Interpret bone mineral density test results and diagnose osteoporosis
- List laboratory tests to order when evaluating for secondary causes of osteoporosis
- Describe lifestyle modifications to prevent and treat osteoporosis
- Describe pharmacologic treatment options for osteoporosis and their potential side effects
- Identify which patients require drug holiday from bisphosphonates

**1) What is osteoporosis? (Annals ITC8)**

Systemic skeletal disorder that is characterized by bone fragility and increased risk for fracture.

**2) What are the USPFTF guidelines for osteoporosis screening? (Annals ITC18)**

All women aged 65 or older should be screened for osteoporosis with dual energy x-ray absorptiometry (DXA).

**3) When would you consider to screen younger women for osteoporosis? (Annals ITC18)**

You may consider to screen post-menopausal women before age 65 if:

- Osteoporosis Self Assessment Tool Score <2 (OST Score = [weight (kg) age (years)] × 0.2)
- 10 year fracture probability per FRAX is ≥ to fracture risk of 65 y/o women without additional risk factors
- Patent has additional risk factors (review risk factors below- ques 5)

**4) How frequently should we screen for osteoporosis? (Annals ITC19)**

No specific guidelines but studies suggest that those with lower BMD should be screened more frequently. Study by Gourlay et al suggest retesting intervals based on baseline BMD:

- Normal BMD (T-score ≥1.0) or mild osteopenia (T-score 1.5): every 15 yrs
- Moderate osteopenia (T-score 2.0): every 5 yrs
- Advanced osteopenia (T-score 2.5): every 1 yr

**5) List at least 5 risk factors for osteoporosis (Annals ITC19- Box)**

Lifestyle: alcohol use, low BMI, smoking, poor nutrition, immobilization, malabsorptive bariatric surgery, gastric bypass surgery, recurrent falls

Comorbidities: Vit D insufficiency, hypercalciuria, celiac disease, cushing syndrome, IBD, diabetes

Hormonal states: premature menopause, premature ovarian insufficiency, panhypopituarism, hyperprolactinemia, androgen insufficiency, hyperthyroidism

Medications: antiepileptics, glucocortoids, GnRH antagonists, SSRI, thiazolidinediones, aromatase inhibitors, lithium

Age, gender, first degree relative

*Talking points: Learners should list at least 3 in each category. Should emphasize that there are certain medications that can decrease bone density*

**6) What is the diagnostic criteria for osteoporosis? (Annals ITC22)**

- BMD T-score at or <-2.5
- History of hip or vertebral fracture not due to excessive trauma
- Hx of radiographic vertebral fx

**7) What’s the difference between the T-score and Z-score? When would you use each? (Annals ITC22)**

T-score is the SD below or above the average BMD of a young adult. This should be used in reporting BMD for post-menopausal women and men>50 y/o.

The Z-score is the SD below or above the average BMD of individuals who are the same age, sex and race as the patient. The Z-score should be used for premenopausal women and men<50 y/o. You cannot diagnose osteoporosis with the Z-score.

**8) What are secondary causes of osteoporosis and what tests can you order to work these up? (Annals ITC23)**

Usually osteoporosis due to old age and low hormone states. There are no current guidelines to support routine lab testing for osteoporosis. Can start w/u with Cr, TSH, Vit D, and Ca level.

- CKD: Cr, Phos, Ca, PTH
- Hyperthyroid: TSH
- Vit D deficiency: 25(OH)-vit D level
- Paget’s Disease: Alkaline phosphatase
- Hyperparathyroid: PTH, Ca, Phos, Vit D
- Autoimmune diseases: ESR, CRP, CBC
- Hypogonadism: Testosterone (men), estradiol (women), LH, FSH
- Hyperprolactinoma: Prolactin level
- Multiple myeloma: SPEP
- Malabsorption, celiac disease: tissue transglutaminase antibody
- Hypercortisol states: Salivary cortisol

*Talking points: Emphasize that test depends on clinical suspicion for secondary cause. Learners should know at least assessing for renal dysfunction, thyroid disease, vitamin D deficiency and hyperparathyroidism.*

**9) What lifestyle recommendations can you provide patents to prevent osteoporosis? (Annals ITC21)**

- Maintain a healthy body weight
- Smoking cessation
- Avoid excessive alcohol use
- Resistance and weight bearing exercises (Ex. Running, walking, weight lifting, etc)- at least 30min/day
- Balance training to avoid falls
- Adequate calcium and vitamin D intake

**10) What is the recommended daily intake of vitamin D and calcium for women? (Annals ITC21)**

| ***Age*** | ***Calcium*** | ***Vitamin D*** |
| --- | --- | --- |
| 19-50 | 1000mg/day | 600 units daily |
| >50 y/o | 1200mg/day | 800 units daily |

Should warn patients that excessive intake of calcium can increase risk of nephrolithiasis.

*Talking points: Note that these are US RDA recommendations*

**11)** **Complete the table below to describe the pharmacologic treatments available for osteoporosis. (NEJM- pg 257)**

Goals of treatment are to prevent fracture because hip fracture is associated with worse quality of life and increased risk of death.

| **Medication** | **Who should receive** | **Administration** | **Mechanism of Action** | **Side Effects/ Contraindications** |
| --- | --- | --- | --- | --- |
| **Bisphosphonates**  PO: Alendronate, risendronate  IV: ibandronate, zoldronic acid | -First line for osteoporosis and prevention  -BMD T score ≤ -2.5 or has existing vertebral fracture  May consider pts who have:  -Osteopenia + FRAX 10 year risk of hip fracture ≥3%  -Osteopenia + FRAX 10 year risk of major osteoporotic fracture ≥20%  -Reduces vertebral, non-vertebral and hip fxs | Oral or IV | Inhibit bone remodeling | SE:  -Esophagitis  -mild hypocalcemia  -MSK pain  -osteonecrosis of jaw (risk 0.001-0.01%)  -atypical femur fx  -Flu-like sx (zoldronic)  Contraindications:  -GFR<35 ml/min  -Vit D deficiency  -Hx of esophageal disease (ex. Achalasia, Barrett’s) |
| **Biologic**: Denosumab  (i.e. Prolia) | Pts with osteoporosis who…  -Couldn’t tolerate PO or IV bisphosphonate  -Hx of renal impairement  Reduce risk of vertebral, non-vertebral and hip fxs | SQ q 6 mo | Binds to the receptor activator of  nuclear factor-κβ ligand (RANKL) and inhibits bone  resorption by binding to | Eczema, cellulitis at injection sites, ONJ, atypical femur fractures  -Must monitor Ca, Mg, Phos 2 weeks after injection |
| **PTH, anabolic**:  teriparatide  (i.e. Forteo), abaloparatide | -Reduce risk of vertebral and nonvertebral fx  -Could not tolerate bisphosphonates  -Had major osteoporotic fx while on bisphosphonates  -Max 2 years (Antiresorptive med should follow) | SQ injection | Increase bone formation | Nausea, arthralgia, leg cramps,  hypercalcemia, hypercalcuria; hyperuricemia, hypotension, osteosarcoma (1 in 1million cases) |
| **SERM**: Raloxifene | Treat and prevent osteoporotic vertebral fx only  Also reduces risk of breast CA | Oral | Activate estrogen receptors leading to reduced bone resorption | Venous thromboembolism, hot flashes, leg cramps, nausea |
| **Estrogen** | **Prevention** of osteoporosis only  When also trying to relieve menopausal sx |  | Affect osteocytes, osteoclasts,  and osteoblasts, leading to inhibition of bone  resorption | Increase risk of breast cancer and coronary,  cerebrovascular, and thrombotic events |
| **Calcitonin** | Reduce vertebral fx only  Could not tolerate bisphosphonates |  |  | Nasal congestion |

ACP recommends offering bisphosphonates or denosumab to patients with osteoporosis. It is AGAINST recommending estrogen therapy or raloxifene.

*Talking points: Should be aware of medication groups and emphasize highlighted points above*

**12) What is the risk of developing osteonecrosis of the jaw from bisphosphonates and what factors increase a patient’s risk? (Annals ITC28)**

Risk is relatively low: 0.001 – 0.01%.

Increased risk factors include: poor oral hygiene, glucocorticoids, chemotherapy, smoking, diabetes, and a recent history of invasive dental procedures

**13) When should patients receive a drug holiday from bisphosphonates? (Annals ITC28)**

Consider a drug holiday if pt has been on PO bisphosphonate for 5 yrs or IV bisophosphonates for 3 yrs and if the patient:

1. Did not have osteoporotic fx before or during treatment period
2. Hip BMD T-score >-2.5 after treatment period
3. Is not at high risk for fracture

Pt should be on drug holiday for 2-3 years.

**References**

- Ensrud, K and Crandall, C. Osteoporosis. *Ann Intern Med*. 2017;167:ITC17–ITC32.
- Black, D. and Rosen, C. Postmenopausal Osteoporosis. *N Engl J Med* 2016;374:254-62.
