# Supplementary material for: Using the Jigsaw Teaching Method to Enhance Internal Medicine Residents' Knowledge and Attitudes in Managing Geriatric Women's Health
Source: MedEdPORTAL. 2020 Oct 23;16:11003. doi: 10.15766/mep_2374-8265.11003 (PMC7586752; doi:10.15766/mep_2374-8265.11003)
Supplement: Supplementary file 1 — Expert Group Reading Materials.docxStudent Worksheet-Group A AUB.docxStudent Worksheet-Group B Osteoporosis.docxStudent Worksheet-Group C Menopause.docxStudent Worksheet-Group D UI.docxStudent Worksheet-Patient Cases.docxFacilitator Guide-Group A AUB.docxFacilitator Guide-Group B Osteoporosis.docxFacilitator Guide-Group C Menopause.docxFacilitator Guide-Group D UI.docxFacilitator Guide-Patient Cases and Debriefing Questions.docxFacilitator Guide Overview and Jigsaw Instructions.docxGeriatric Women's Health for IM Residents.pptxPretest.docxPosttest.docx [file mep_2374-8265.11003-s001.zip › I. Facilitator Guide-Group C Menopause.docx]

***Note to Facilitators:*** *During the expert group activity, please circulate the room to ensure that learners are discussing accurate teaching points. You should provide immediate feedback if discussions are off topic or information is incorrect. This document will include take home points for each question but it is not meant to provide to learners verbatim. During expert review, learners should provide answers to you first and then you can fill in gaps. We included detailed answers in case you are not as familiar with this topic and have one comprehensive reference for the topic.*

**Learning Objectives**

- Define menopause and when it typically occurs in women
- Identify factors that affect the onset of menopause
- List the stages of menopause
- Describe symptoms associated with menopause and how the diagnosis of menopause is made
- Describe lifestyle modifications to treat the common menopause symptoms
- Describe the indications, side effects, and contraindications of hormone replacement therapy
- List and describe non-hormonal medication options for managing menopause
- Describe the complementary-alternative medicine options for menopause symptom relief

**1) What is menopause? (ITC 4-2)**

Cessation of the menstrual cycle and the end of a woman’s reproductive years. Defined RETROSPECTIVELY 12 months after final menstrual period.

Menopause is primary ovarian failure. There is ovarian follicle depletion most likely secondary to apoptosis or programmed cell death. The ovary no longer responds to the pituitary hormones FSH and LG and thus ovarian estrogen and progesterone production decreases.

*Talking points: No period x 12 months*

**2) When does menopause occur? (ITC 4-2)**

Median age 51.3 years. Irregular menses mark the transition into menopause and begin 4 years before final menstrual period.

*Talking points: On average when women turn 50 but can be earlier or later*

**3) What factors affect age of onset of menopause? (ITC 4-2)**

Age at menopause appears to be genetically determined but can be influenced by other factors. Smokers have earlier onset (median age 50.2 years).

Premature menopause can also occur with hysterectomy/oophorectomy/chemo exposure/pelvic XRT exposure (even if hysterectomy with ovaries left in place, menopause can still occur early).

**4) What are the stages of menopause? (ITC 4-2, Table 1)**

STRAW-Stages of Reproductive Aging workshop describes menopause as series of distinct stages.

Early perimenopause- irregularity in menstrual cycle due to anovulatory cycles (irregular length, duration). Late perimenopause-progressive irregularity which ends 1 year after final period.

Postmenopause is either early (first 4 years after final period) or late (5+ years).

*Talking points: Periods will start to become irregular, lighter and shorter and then ultimately stop.*

**5) What symptoms suggest menopause and over what time period do they occur? (ITC 4-2, ITC 4-3)**

Symptom complexes follow a somewhat predictable pattern over a highly variable time course. Vary amongst individuals in timing, prevalence, severity, duration.

1. vasomotor symptoms* (most common, affects up to 75% women; typically peak by final menstrual period and then decrease in prevalence/severity; last 1-2 years in many women but can continue in others for 10+ years)

- hot flash or flush-sudden, intense warmth, begins face/chest and spreads throughout body (can have diaphoresis + palpitations); variable time 2-30 minutes; can disrupt sleep at night

1. Urogenital atrophy- vaginal dryness symptoms/discomfort/pruritic/painful intercourse (affects 1/3-1/2 of women, symptoms increase with age)
2. Sleep disturbances (may be related to vasomotor symptoms)
3. Mood disorders (depression, anxiety, irritability)
4. Cognitive disturbances
5. Somatic complaints (back pain, stiff/painful joints/tiredness/myalgia)
6. Urinary incontinence
7. Sexual dysfunction not related to dyspareunia

#1 and 2 definitely associated with menopausal hormonal changes, others not definitely related.

**6) What are the diagnostic criteria for menopause? (ITC 4-3)**

Obtaining history of menstrual changes and typical menopausal symptoms is usually sufficient to diagnose menopause. Testing rarely needed.

Lab tests should be considered if diagnosis unclear, if atypical symptoms present, or if premature ovarian failure suspected (before age 40). FSH can confirm menopause (elevated level >30mIU/mL) is objective evidence that symptoms related to menopause (the ovarian-hypothalamic-pituitary axis remains intact during menopause thus FSH rises in response to ovarian failure and absence of negative feedback from ovary).

*Talking points: Highlight that this is a clinical diagnosis but can be confirmed with lab testing*

**7) What lifestyle modifications can relieve menopausal symptoms? (ITC 4-4)**

- Decrease cigarette use or Quit smoking (smoking associated with worsening of vasomotor symptoms)
- Decreasing amount of alcohol intake or abstain (alcohol is a vasodilator and may be associated with vasomotor symptoms)
- Exercise-data lacking on reduction of symptoms with exercise but it does favorably affect mood, stress, body image.
- Weight loss (overweight women have more severe vasomotor symptoms)
- Dress in layers, use fan, avoid hot drinks/caffeine/hot and spicy food, use ice packs
- Use vaginal moisturizer or lubricants for vaginal dryness
- Kegel exercises for stress urinary incontinence

*Talking points: Should know this information thoroughly to provide patient education*

**8) What hormonal treatment is available for menopause? What is the dosing and duration of therapy? (ITC 4-9)**

- HRT is usually estradiol or conjugated equine estrogen. If woman still has a uterus, should use combination hormone therapy (estrogen with progestin) to avoid increased risk for endometrial cancer.
- Need to weigh benefits and risk
  - Benefits= vasomotor and urogenital relief; reduction in future risk for osteoporosis and colorectal cancer
  - Risks= increased risk for venous thromboembolism, cardiovascular disease, breast cancer
- Vaginal administration of estrogen treats vaginal atrophy but symptom relief can take months and vaginal estrogen can have systemic side effects
- Treat for shortest possible time at the lowest effective dose.
- Frequently reassess symptoms and ask about unexpected bleeding or adverse effects.

**9) What are side effects of HRT and what should be monitored when a patient is on HRT? What are contraindications of prescribing HRT? (ITC 4-7 to ITC 4-8)**

Most common acute adverse effect with estrogen therapy: breast tenderness and uterine bleeding, nausea and vomiting, headache, weight change, dizziness, VTE, cardiovascular events, liver effects, rash and pruritis.

Monitor: Age appropriate screening including that for breast and colorectal cancer, Bone Mineral Density every 2 years to assess osteoporosis, lipid panel

ABSOLUTE CONTRAINDICATIONS to HRT: pregnancy, unexplained vaginal bleeding, active or chronic liver disease, acute cardiovascular disease, history of cardiovascular disease, history of breast or endometrial cancer, history of VTE related to hormone therapy, recent thrombosis, hypertriglyceridemia.

*Talking points: See highlight above*

**10) What non-hormonal treatments are available for menopause? (ITC 4-10, Table 6)**

Clonidine and some antidepressants and anticonvulsants (gabapentin) can improve vasomotor symptoms.

Complementary-alternative treatments:

Studies tend to be small, short duration, and in selected samples.

Studies on botanical black cohosh as well as soy have had mixed results.

(Others that do not seem to be effective include Dong quai root, kava, St Johns wort, red clover, evening primrose, ginseng).

*Talking points: Mostly review studied SSRI’s (see table 6 in article)*

**RESOURCES**:

Nananda F. Col, Kathleen M. Fairfield. Osteoporosis. *Ann Internal Med.* 2009; 4:ITC2-16
