# Supplementary material for: Using the Jigsaw Teaching Method to Enhance Internal Medicine Residents' Knowledge and Attitudes in Managing Geriatric Women's Health
Source: MedEdPORTAL. 2020 Oct 23;16:11003. doi: 10.15766/mep_2374-8265.11003 (PMC7586752; doi:10.15766/mep_2374-8265.11003)
Supplement: Supplementary file 1 — Expert Group Reading Materials.docxStudent Worksheet-Group A AUB.docxStudent Worksheet-Group B Osteoporosis.docxStudent Worksheet-Group C Menopause.docxStudent Worksheet-Group D UI.docxStudent Worksheet-Patient Cases.docxFacilitator Guide-Group A AUB.docxFacilitator Guide-Group B Osteoporosis.docxFacilitator Guide-Group C Menopause.docxFacilitator Guide-Group D UI.docxFacilitator Guide-Patient Cases and Debriefing Questions.docxFacilitator Guide Overview and Jigsaw Instructions.docxGeriatric Women's Health for IM Residents.pptxPretest.docxPosttest.docx [file mep_2374-8265.11003-s001.zip › J. Facilitator Guide-Group D UI.docx]

***Note to Facilitators:*** *During the expert group activity, please circulate the room to ensure that learners are discussing accurate teaching points. You should provide immediate feedback if discussions are off topic or information is incorrect. This document will include take home points for each question but it is not meant to provide to learners verbatim. During expert review, learners should provide answers to you first and then you can fill in gaps. We included detailed answers in case you are not as familiar with this topic and have one comprehensive reference for the topic.*

**Learning Objectives**

- Define urinary incontinence (UI)
- Describe the prevalence and costs of UI
- Describe the effects that UI has on an individual’s life
- Describe how micturition is controlled and list the different types of incontinence that can develop
- List risk factors for developing incontinence
- Describe the evaluation and work-up of incontinence
- Describe nonsurgical treatments available for incontinence including those recommended lifestyle modifications
- List pharmacologic options for incontinence and describe the adverse effects associated with these treatments

**1. What is Urinary Incontinence? (AFP page 634)**

ANY involuntary leakage of urine, loss of bladder control.

**2. How prevalent is UI and how does it affect the patient and society? (AFP page 634)**

- 20 Million people are affected by incontinence, prevalence ranges from 3-55% based on type of incontinence and population being examined. In nursing homes, rates are more near 60-70%. Note: at least half of patients do not report incontinence symptoms to their physician therefore these estimates are on the conservative end (and thus varied per study)
  - Some sources cite approximately 30% of women suffer from some degree. 10% of women suffer weekly incontinence, 5% have daily incontinence.
- Incidence increases with age. Overweight and obese women more likely to report symptoms.
- Incontinence greatly affects the patient- it can dramatically alter a woman’s life because of leakage, curtailing activities for fear of loss of control, and change in intimacy due to symptoms. Many patients have a deterioration in their self-esteem.
- The total annual cost to care for patients with incontinence is estimated at $11.2 billion in the community and $5.2 billion in nursing homes.

*Talking points*: *Highlight that UI is very common and costly on health system*

**3. What factors control micturition? (Berek Page 862)**

- Bladder storage and emptying depends on a complex interplay between the brain, spinal cord, bladder, urethra, and pelvic floor.
- There are intrinsic factors that contribute to urethral closure and extrinsic urethral support.

**4. What types of urinary incontinence are there? (AFP page 635, Table 1)**

- Most common types: stress, urge, and mixed. Overflow incontinence (due to urinary retention) and functional incontinence (due to inability to get to toilet due to physical or cognitive limitations) exist but are far less common. Main focus of this topic will be on the three more common types.
- Stress (occurs with effort or exertion such as increases in abdominal pressure- coughing, running, lifting)-*Most common form in women, particularly younger women
  - Occurs when intravesical pressure rises higher than the pressure that the urethral closure mechanism can withstand.
- Urge (occurs with sudden sense of urgency)-most common form in older women
- Mixed (associated with urgency and also with effort or physical exertion or on sneezing or coughing)

*Talking points*: *Should be able to tell difference between stress vs urge vs mixed incontinence*

**5. What are the risk factors for incontinence? (Berek Page 871)**

- Age, pregnancy, childbirth, obesity, functional impairment, and cognitive impairment are associated with increased rates of incontinence or incontinence severity.
- Pregnancy and delivery predispose women to stress urinary incontinence during younger years.
- Medical conditions such as diabetes, CVAs, spinal cord injuries.

**6. What is the work-up and evaluation for urinary incontinence? (Berek, page 871-877)**

Key historical questions: how often is urine leaked, how much urine is leaked, what provokes the leakage, what improves or worsens the problem, and what treatment may she have had in the past? What medications is that patient taking?

There are validated questionnaires to assess symptoms and effect on quality of life.

Please refer to Table 26.3: Medications that May Affect the Urinary Tract in Menefee, S. and Nygaard, I. 2012. “Chapter 26 Lower Urinary Tract Disorders.” Ed. J. Berek and D. Berek. Philadelphia: Lippincott Williams & Wilkins, 2012. 862-874, 884-888.

- Voiding/Bladder diary- frequency and volume chart-documents every void, any incontinent episodes, related activities, can also track fluid intake. *This diary gives the clinician a lot of information that cannot be provided by formal testing🡪 24 hour urine output, total number voids, number nighttime voids, voided volumes, and functional bladder capacity.
- Urinalysis- to rule out infection, hematuria, and/or metabolic abnormalities
- Post void residual (assess incomplete bladder empyting as this can cause incontinence)
- Cough stress test
- Pad tests-weighing menstrual or bladder pads before and after activity can objectively measure urine leakage

Advanced testing includes: urodynamics, uroflowmetry, filling cystometry

*Talking points*: *Highlight importance of medication review and to rule out infection.*

**7. What nonsurgical treatment is available for urinary incontinence? (AFP page 635: table 2, AFP page 636: table 3)**

Approach to treatment is a stepped care plan starting with noninvasive behavioral modifications then followed by pharmacologic interventions and finally devices/surgery as a last resort for those not responding to initial efforts (sacral nerve stimulator for urge incontinence, sling procedures or urethropexy for stress incontinence)

1. Lifestyle changes:
   1. Weight loss
   2. Postural changes (i.e. crossing legs during increased intra-abdominal pressure)
   3. Decreasing caffeine, monitoring fluid intake in general
2. Physical therapy-pelvic floor muscle training (Kegel exercises) for stress and urge incontinence (many think of this only for stress type incontinence but it is effective in urge type incontinence and can be more effective than medications)
3. Behavioral therapy and bladder training for urge incontinence (modifying bladder function by changing voiding habits; improving voluntary control rather than bladder function- scheduled toileting program)
   1. Review patient voiding diary and choose an interval that represents the longest interval between voiding that is comfortable and pt instructed to void every time that interval passes. If patient feels the urge to void before the time, patient to use urge suppression strategies to get to stated interval. Also, scheduled voiding or prompted voiding.
4. Vaginal devices, i.e. pessaries and urethral inserts

Please refer to Table 3: Common Behavioral Therapy for UI Hersh, L and Salzman, B. Clinical Management of Urinary Incontinence in Women. *American Family Physician*. 2013; 87(9): 635-640.

*Talking points*: *As primary care providers, can recommend lifestyle modification, PT and behavior therapy.*

**8. What medications are used for the treatment of urinary incontinence? (Berek Page 888 Table 26.9, AFP page 635: Table 2)**

1. Stress incontinence-No drugs are cleared by the FDA to treat stress incontinence. The tone of the urethra and bladder neck is maintained by alpha-adrenergic activity this some meds with this effect have been tried but none approved. Cymbalta is another medication mentioned in articles, but not FDA approved for this use. If lifestyle changes and other nonsurgical treatment is not effective, surgery may be required.
2. Urge incontinence-drugs for detrusor overactivity- ANTICHOLINERGIC agents. Other options include a beta-adrenergic agonist (Myrbetriq) and botox. Intravaginal estrogen is noted in articles but is not FDA approved for this use.

Please refer to:

- Table 26.9: Commonly used medications in UI in Menefee, S. and Nygaard, I. 2012. “Chapter 26 Lower Urinary Tract Disorders.” Ed. J. Berek and D. Berek. Philadelphia: Lippincott Williams & Wilkins, 2012. 862-874, 884-888
- Table 2: Common Medications for UI Hersh, L and Salzman, B. Clinical Management of Urinary Incontinence in Women. *American Family Physician*. 2013; 87(9): 635-640.

*Talking points*: *More medications for urge incontinence but not much for tress incontinence.*

**9. What are the side effects of the medications for urge incontinence? AFP page 637**

Since anticholinergic, side effects include dry mouth, increased heart rate, blurred vision, tendency toward overheating, delirium, constipation.

*Because of adverse effects, American Geriatrics Society recommends avoiding unless no other option.

Myrbetriq is a beta adrenergic agonist and its side effects include: nausea, diarrhea, constipation, dizziness, headache, elevated blood pressure.

**RESOURCES:**

- Hersh, L and Salzman, B. Clinical Management of Urinary Incontinence in Women. *American Family Physician*. 2013; 87(9): 635-640.
- Menefee, S. and Nygaard, I. 2012. “Chapter 26 Lower Urinary Tract Disorders.” Ed. J. Berek and D. Berek. Philadelphia: Lippincott Williams & Wilkins, 2012. 862-874, 884-888.
