# Supplementary material for: Using the Jigsaw Teaching Method to Enhance Internal Medicine Residents' Knowledge and Attitudes in Managing Geriatric Women's Health
Source: MedEdPORTAL. 2020 Oct 23;16:11003. doi: 10.15766/mep_2374-8265.11003 (PMC7586752; doi:10.15766/mep_2374-8265.11003)
Supplement: Supplementary file 1 — Expert Group Reading Materials.docxStudent Worksheet-Group A AUB.docxStudent Worksheet-Group B Osteoporosis.docxStudent Worksheet-Group C Menopause.docxStudent Worksheet-Group D UI.docxStudent Worksheet-Patient Cases.docxFacilitator Guide-Group A AUB.docxFacilitator Guide-Group B Osteoporosis.docxFacilitator Guide-Group C Menopause.docxFacilitator Guide-Group D UI.docxFacilitator Guide-Patient Cases and Debriefing Questions.docxFacilitator Guide Overview and Jigsaw Instructions.docxGeriatric Women's Health for IM Residents.pptxPretest.docxPosttest.docx [file mep_2374-8265.11003-s001.zip › K. Facilitator Guide-Patient Cases and Debriefing Questions.docx]

**For facilitators**: Once home groups have worked through patient cases, use the Kahoot! Quiz as a debriefing exercise. Below are the correct answers for the Kahoot! quiz, facilitator talking points, and patient case worksheets. Using the Kahoot! App is optional.

**Kahoot! Debriefing Questions & Answers**

*Instructor Login:* [*https://create.kahoot.it*](https://create.kahoot.it)

*Student Login:* *Plug pin number into* [*https://kahoot.it*](https://kahoot.it)

1. Case 1- What is the cause of Mrs. Johnson's urinary symptoms?
   1. Stress incontinence
   2. **Urge incontinence**-
   3. Mixed incontinence
   4. Urinary tract infection

*Talking Points: Patient has urge incontinence because she had constant urge to urinate and is not leaking urine when coughs or sneezes, which rules out answers A + C. Patient did not have UTI because UA was negative and patient did not have dysuria.*

1. What lifestyle modification would help her urinary symptoms?
   1. **Weight loss**
   2. **Smoking cessation**
   3. **Monitoring fluid intake**
   4. **Schedule bathroom breaks**

*Talking Points: Can discuss other recommendations for lifestyle modifications (ex. Decrease caffeine/alcohol/total liquid intake, etc) and Kegel exercises.*

1. What medication changes may help her urinary symptoms?
   1. Starting anticholinergic agents (ex. oxybutynin)
   2. Stopping cetirizine
   3. Starting beta-adrenergic agents (ex. mirabegron)
   4. **All of these may help**

*Talking Points: Anticholinergic is also anti-muscarinic. All antihistamines can affect urination.*

1. What tests would you order to work up her hot flashes?
   1. TSH
   2. PPD
   3. LH, FSH
   4. **None- she is likely perimenopausal**

*Talking Points: Patient is likely perimenopausal given irregular periods and is in correct age range. Consider work-up if symptoms did not fit or patient is young (<40 years old). Review menopause symptoms including vasomotor, sleep, urogenital symptoms, mood and cognition, incontinence and other sexual dysfunction*

1. What lifestyle modification could help her hot flashes?
   1. **Smoking cessation**
   2. **Weight loss**
   3. Drinking more alcohol
   4. **Avoiding constipation**

*Talking Points: Ask for additional recommendations, such as layering, exercise. Note that decrease alcohol intake improves symptoms.*

1. Which medications would you recommend to help her menopausal symptoms?
   1. Hormone replacement therapy
   2. Vaginal estrogen cream
   3. **Venlafaxine**
   4. **Vaginal lubricant**

*Talking Points: Avoid hormone therapy (oral or topical) in this patient due to history of smoking, family history of breast cancer, and high cardiac risk. Can discuss other side effects of hormonal therapy. Review other medications, including clonidine, gabapentin, black cohosh and soy. Also review how HRT is prescribed (ex. Patient with uterus should have combined HRT, start low and slow, etc)*

1. Which is NOT a contraindication for hormone replacement therapy?
   1. History of VTE
   2. Cardiovascular disease
   3. Chronic liver disease
   4. **Diabetes**

*Talking Points: Other contraindications are hyperlipidemia (high triglycerides), GYN cancers, and thrombosis.*

1. What herbal supplement can sometimes help with hot flashes?
   1. **Black cohosh**
   2. Ginseng
   3. St John’s wort
   4. **Soy**

*Talking Points: Limited effectiveness and should be cautious of side effects of both supplements. Studies did not show benefit of ginseng or St John’s wort. Review side effects of black cohosh and soy.*

1. Case 2- Which of the following increases Mrs. Bailey's risk for osteoporosis?
   1. **Menopause**
   2. **Low BMI**
   3. **Inactivity**
   4. **Phenytoin**

*Talking Points: Other risk factors for patient case- COPD with steroid use, nulliparity. Review other risk factors for osteoporosis: Smoking, malabsorptive diseases, certain medications (ex. steroids, lithium, anticonvulsants, aromatase inhibitors, GNRH antagontists), family history, alcohol*

1. What is her daily recommended calcium intake?
   1. 500mg
   2. **1200mg**
   3. 1600mg
   4. 2200mg

*Talking Points: Review calcium and vit D recommendations based on age:*

| ***Age*** | ***Calcium*** | ***Vitamin D*** |
| --- | --- | --- |
| 19-50 | 1000mg/day | 600 units daily |
| >50 y/o | 1200mg/day | 800 units daily |

1. What is her daily recommended vitamin D intake?
   1. 100IU
   2. 500IU
   3. **800IU**
   4. 2000 IU
2. Based on Mrs. B’s DEXA, she has…?
   1. Normal bone density
   2. Osteoporosis
   3. **Osteopenia**
   4. Paget’s Disease

*Talking Points: Discuss when to start DEXA screening, including age 65, if they are high risk or have had fracture. Review differences between T or Z scores.*

1. Which of the following exercises would help Mrs. B improve her bone health?
   1. **Walking**
   2. **Resistance training**
   3. **Dancing**
   4. Swimming

*Talking Points: Review weightbearing exercises and other lifestyle modifications to improve bone health.*

1. Could Mrs. B start a bisphosphonate?
   1. **Yes**
   2. No
   3. Not sure

*Talking Points: Review indications for bisphosphonates for patients with osteopenia (Ex. FRAX 10 year risk of hip fracture ≥3% (hers was 3.9); FRAX 10 year risk of major osteoporotic fracture ≥20%)*

1. What medication would you offer for osteopenia if she had a history of Barrett's esophagus?
   1. Bisphosphonate
   2. Denosumab (Prolia)
   3. Hormone replacement therapy
   4. **None- only lifestyle modifications**

*Talking Points: Review side effects of bisphosphonates including esophagitis, osteonecrosis of jaw, atypical of femur and contraindications (ex. CKD). Can review that if patient had osteoporosis, can consider denosumab given Barrett’s.*

1. Which of the following could be a cause of her vaginal bleeding?
   1. **Uterine polyp**
   2. Von Willibrand Disease
   3. Hypothyroidism
   4. **Endometrial cancer**

*Talking Points: May also consider fibroid. Review PALM-COEIN acronym to help with differential of AUB.*

1. What are Mrs. B's risk factors for endometrial cancer?
   1. Smoking history
   2. **Age**
   3. **Family history of breast cancer**
   4. **Nulliparty**

*Talking Points: Review other risk factors for AUB- ex. Infertility, obesity, DM, FHx of breast/endo/colon CA, unopposed estrogen use, tamoxifen*

1. What diagnostic tests would you order to work up her AUB?
   1. **Transvaginal US**
   2. **Endometrial biopsy**
   3. CT abdomen and pelvis
   4. Serum beta-HCG

*Talking Points: Looking for endometrial lining thickness with US and there should be a biopsy if >5mm. CT is not helpful.*

1. If her endometrial biopsy showed hyperplasia without atypia, what are her treatment options?
   1. Combined OCP
   2. **Progestin-only therapy**
   3. **Hysterectomy**
   4. Myomectomy
2. What are contraindications to progestin therapy?
   1. **Liver disease**
   2. **Breast cancer**
   3. **Pregnancy**
   4. **Ischemic heart disease**

**Patient Case Worksheet Answers**

**Case 1**- Mrs. Johnson is a 48 year old female with past medical history diabetes, hypertension, and allergic rhinitis who presents with difficulty sleeping. Her symptoms started a few months ago where she would have trouble staying asleep all night due to episodes of feeling extremely hot and sweaty. She has tried layering clothes, opening windows and using fans in her room but nothing seems to help. Her sleep is also disturbed by her constant urge to go to the bathroom. This increased urgency also occurs during the day and feels that she runs to the bathroom every hour. She endorses some vaginal dryness, constipation, but denies any fever, weight change, leaking of urine with coughing, pain with urination, or blood in her urine.

Past Medical History: type 2 diabetes, hypertension, allergic rhinitis

Past Surgical History: None

Gynecological History:

- LMP: 3 months ago, has been every few months over the past year
- 3 pregnancies. All vaginal deliveries
- Sexual hx- 1 sexual partner, admits to some pain with intercourse
- Last pap smear- 2 years ago, no history of abnormal
- Mammogram- 1 year ago, no history of abnormal

Medication: Metformin, Lisinopril, cetirizine

Family History: Mom- DM; Dad- CAD; no family history of cancer or blood clots

Social History: Smokes 5 cigarettes per day. Drinks a glass of wine 3-4 times a week with dinner

Physical Exam is only notable for BMI 35 and vaginal atrophy

In office U/A: negative

**What are the likely causes for this patient’s symptoms? What would you recommend to her to provide relief? If you are prescribing medication, what side effects would you educate her on?**

*Mrs. Johnson is likely experiencing perimenopausal symptoms and urge incontinence. You can recommend the following:*

- *Urge Incontinence*
  - *Lifestyle modifications*
    - *Weight loss*
    - *Smoking cessation*
    - *Avoiding constipation*
    - *Bladder training- urge suppression*
  - *Medications:*
    - *Should consider stopping cetirizine since antihistamines may impair detrusor contractility*
    - *Anticholinergic agents (Ex. Oxybutynin, vesicare)*
      - *Side effects include dry mouth, increased HR, blurred vision, heat intolerance, constipation*
    - *Beta-adrenegic agent (Ex. Mirabegron/Mybetriq)*
- *Menopausal symptoms- hot flashes, vaginal dryness/atrophy*
  - *Lifestyle modifications*
    - *Smoking cessation*
    - *Decrease alcohol intake*
    - *Weight loss*
    - *Avoid hot drinks and spicy food*
    - *Use fans*
    - *Apply ice packs*
  - *Medications*
    - *CANNOT use HRT given her hx of smoking*
    - *Vaginal moisturizer/lubricant*
    - *Trial SSRI- paroxetine or venlafaxine (SE: Dry mouth, nausea, drowsiness, constipation)*

**Case 2**- Mrs. Bailey is a 67 y/o widowed female with a history COPD with frequent exacerbations, hyperlipidemia, epilepsy, and past tobacco use who presents to review her recent labs and bone density test. She overall feels well but upon review of symptoms, she mentions intermittent vaginal spotting over the past month. It has occurred at least once weekly and persists for 2-3 days. She denies any vaginal pain, recent intercourse, weight loss, abdominal pain, dysuria, hematuria, or bloody bowel movements. She has never had any issues bleeding in the past, including never having prolong bloody nose, gum bleeding or joint bleeding.

Past Medical History: COPD, epilepsy, hyperlipidemia, wrist fracture 2 years ago. She has been in the hospital 4 times this past year for COPD exacerbations.

Past Surgical History: Appendectomy- no complications

Gynecologic history:

- Menopause since age 52
- No history of pregnancy
- Sexual history- no current partners. Husband passed away last year and they were married for 35 years
- Last pap smear- stopped after age 65, no history of abnormal paps

Medications: fluticasone/salmeterol inhaler, tiotropium inhaler, phenytoin, pravastatin, aspirin

Family History: Mom- breast cancer, osteopenia; Dad- HTN, emphysema

Social history: Retired waitress. Spends most of the day sitting. Used to smoke 1 pack per day for 40 years. Quit last year after she was hospitalized. Social drinkers- 1-2 drinks every few weeks

Physical Exam notable for:

- BMI 18.3, Ht 5’5”, Wt 105lbs, BP 100/70, HR 90
- HEENT- Pale conjunctiva
- Lungs- Distant lung sounds, prolong expiration
- Bimanual exam- unremarkable, no blood in vaginal vault
- Remainder exam normal

Labs:

- CBC- WBC 8.2, Hg 9.5, Hct 29.2, Plt 224
- BMP- Na 135, K 4, Cl 105, CO2 25, BUN 22, Cr 1.0
- LFT- AST 23, ALT 26, AP 88, TP 7.2, TB 1.1
- TSH 0.7
- INR 1.3
- Vitamin D level 32

DEXA Results:

| **Location** | **T-Score** | **Z-score** | 10-year risk of major osteoporotic fracture = 16%  10-year risk of hip fracture = 3.9% |
| --- | --- | --- | --- |
| Trochanter | -2.0 | -2.5 |  |
| Femoral neck | -1.9 | -2.3 |  |
| L1-L4 | -0.77 | -0.57 |  |

1. **What risk factors does this patient have for osteoporosis?**

*Ms. Bailey’s risk factors for osteoporosis include:*

- *Low BMI*
- *Post-menopause*
- *Smoking history*
- *Lack of weight bearing exercises*
- *Medications- phenytoin, possible extensive exposure to corticosteroids (frequent COPD exacerbation)*
- *Nullparity*

1. **What is your interpretation of her bone density test? What would you recommend to this patient regarding her bone health? If you are prescribing a medication, how long will she need to be on the medication and what side effects would you educate her about?**

*Ms. Bailey has moderate osteopenia. We would recommend:*

- *Adequate daily calcium (1200mg/day) and vitamin D (800 IU/day) intake*
- *Weight-bearing exercises and resistance training*
- *Discussion about starting a bisphosphonate since her 10-year risk of a hip fracture is >3%. She would be on the medication for max of 5 years.*

*If she agrees to start an oral bisphosphonate, we would need to tell her to take the medication on an empty stomach with a full glass of water. She cannot lay flat for at least 1 hour after taking the medication because bisphosphonates are known to cause esophagitis. Other side effects include: muscle pain, osteonecrosis of the jaw and atypical femur fracture*

1. **What is your assessment of her vaginal bleeding? What would be your next steps?**

*Patient has abnormal uterine bleeding and we should be most concerned for malignancy given that she is post-menopausal. Her other risk factors for endometrial cancer include: age>50, nulliparity, and family history for breast CA. Her bleeding may be significant too because she has evidence of anemia. Other potential diagnoses include structural abnormalities such as polpy, uterine fibroid, or endometrial hyperplasia*

*To work up her AUB, we would order a transvaginal US and refer to GYN for an endometrial biopsy.*

1. **If her endometrial biopsy returned as hyperplasia without atypia, what would her treatment options include?**

*This patient could get a hysterectomy or could undergo progestin therapy.*
