# Supplementary material for: Using the Jigsaw Teaching Method to Enhance Internal Medicine Residents' Knowledge and Attitudes in Managing Geriatric Women's Health
Source: MedEdPORTAL. 2020 Oct 23;16:11003. doi: 10.15766/mep_2374-8265.11003 (PMC7586752; doi:10.15766/mep_2374-8265.11003)
Supplement: Supplementary file 1 — Expert Group Reading Materials.docxStudent Worksheet-Group A AUB.docxStudent Worksheet-Group B Osteoporosis.docxStudent Worksheet-Group C Menopause.docxStudent Worksheet-Group D UI.docxStudent Worksheet-Patient Cases.docxFacilitator Guide-Group A AUB.docxFacilitator Guide-Group B Osteoporosis.docxFacilitator Guide-Group C Menopause.docxFacilitator Guide-Group D UI.docxFacilitator Guide-Patient Cases and Debriefing Questions.docxFacilitator Guide Overview and Jigsaw Instructions.docxGeriatric Women's Health for IM Residents.pptxPretest.docxPosttest.docx [file mep_2374-8265.11003-s001.zip › L. Facilitator Guide Overview and Jigsaw Instructions.docx]

**Facilitator Guide: Session Overview and Jigsaw Instructions**

***Preparation for facilitators***

- Read all expert group review articles
- Print student worksheets (Appendices B to F)
- Print facilitator guides (Appendices H to K)
- Bring extra pens/pencils for learners
- Send reminder to learners at least 1 day prior to session asking them to
  - Bring an electronic device to participate in Kahoot!, such as a smart phone, tablet or laptop
  - Bring a pen/pencil
  - *Optional*: Read their assigned topic ahead of time. If facilitators do not want learners to pre-read, then this part can be eliminated
- Room set up:
  - Group chairs +/- tables into home groups. You can make signs around the room to direct learners
  - Ensure that there is a computer and a large display, such as a projector with a screen or large monitor

***Jigsaw Teaching Method Steps***

1. **Learners complete a pretest.**
2. **Learners break into groups of 4. This will be considered their “Home Group.”**
    To make home groups, facilitators can pre-assign learners or create groups at the beginning of the session. Facilitators will divide learners into groups of 4 based on the total number of participants. If the total number does not divide evenly by 4, facilitators should split remaining learners evenly across previous created groups of 4. For example- A classroom has a total of 23 learners. The session will have 2 home groups of 4 and 3 home groups of 5.

1. **Each member in the home group will be assigned A/B/C/D. Each letter is associated with becoming an “expert” in specific topic (see below). In this geriatric women’s health workshop, the expert topics were:**

   **A = Abnormal Uterine Bleeding (AUB)
   B = Osteoporosis
   C = Menopause
   D = Urinary Incontinence**For home groups with more than 4 learners, facilitators will assign 2 learners to one topic. Our educators favored assigning 2 learners to topics A and D since these topics had longer readings

A

D

B

C

B

A

C

D

A

B

B

A

D

D

C

C

1. **Learners break into their “Expert Groups” to learn their assigned topic.** Learners will have 30 minutes to complete topic-specific worksheets with the review articles given to them. Facilitators should circulate the room periodically to ensure that learners are sharing appropriate teaching points and can immediately provide feedback to keep learners on task. Facilitators should give annoucements with how much time is left in their activity (ex. 15 minute and 5 minute warnings).
    After learners finish their worksheets, facilitators should take 10 minutes to verbally review worksheet answers with each expert group and emphasize talking points of for each expert group. Worksheet answers and talking points are provided in each topic facilitator guide (Appendices G to J).

B: Osteoporosis

**B**

**B**

**B**

**B**

**A**

**A**

**A**

**A**

A: AUB

**D**

**D**

**D**

**D**

D: UI

C: Menopause

**C**

**C**

**C**

**C**

1. **Learners return to their home-groups to peer teach their topics and collective apply their knowledge to solve 2 patient cases.** Learners have 40 minutes to complete the 2 patient cases in their home groups. Each case highlights main teaching points for each topic and peers with teach each their topics through the case. Facilitators should circulate around the room again to listen to discussion between groups and keep track of feedback to be reviewed during the final debrief session. Facilitators can answer questions from the learners. Facilitators again should provide time warnings to ensure the session runs on time (ex. 20 minute, 10 minute and 5 minute warnings).

**A**

**C**

**D**

**B**

**A**

**C**

**D**

**B**

**A**

**C**

**D**

**B**

**A**

**C**

**D**

**B**

Each jigsaw group has at least one topic expert for every topic

1. **Facilitator(s) will then review case answers and debrief with the entire group to reinforce learning objectives. This workshop used the Kahoot! educational platform to facilitate discussion. The questions in the Kahoot! game were from the case (See Appendix K). This electronic platform is optional.**

D

Facilitator

A

A

A

A

B

B

B

B

C

C

C

C

D

D

D

When going through case questions, facilitators should have learners provide their clinical reasoning and elaborate on why they chose a particular answer. Depending on answers, facilitators will provide feedback and fill education gaps.

If facilitators do not want to use Kahoot!, they can simply show a PowerPoint with the case questions and verbally review answers with the group. They may chose individual learners or teams to collectively provide answers. If facilitators want to continue the debriefing as a game, facilitators can pose the questions to each team and whoever raises their hand first would be selected to answer. To gain points, teams must answer the question correctly and additional points can be given to the team who answered correctly first.

1. **Learners complete a post-test to assess if learning objectives were achieved.**

**References**

- The Jigsaw Classroom- <https://www.jigsaw.org/>
- Kahoot! <https://kahoot.com/>
