# Supplementary material for: Using the Jigsaw Teaching Method to Enhance Internal Medicine Residents' Knowledge and Attitudes in Managing Geriatric Women's Health
Source: MedEdPORTAL. 2020 Oct 23;16:11003. doi: 10.15766/mep_2374-8265.11003 (PMC7586752; doi:10.15766/mep_2374-8265.11003)
Supplement: Supplementary file 1 — Expert Group Reading Materials.docxStudent Worksheet-Group A AUB.docxStudent Worksheet-Group B Osteoporosis.docxStudent Worksheet-Group C Menopause.docxStudent Worksheet-Group D UI.docxStudent Worksheet-Patient Cases.docxFacilitator Guide-Group A AUB.docxFacilitator Guide-Group B Osteoporosis.docxFacilitator Guide-Group C Menopause.docxFacilitator Guide-Group D UI.docxFacilitator Guide-Patient Cases and Debriefing Questions.docxFacilitator Guide Overview and Jigsaw Instructions.docxGeriatric Women's Health for IM Residents.pptxPretest.docxPosttest.docx [file mep_2374-8265.11003-s001.zip › M. Geriatric Women's Health for IM Residents.pptx]

## Slide 1
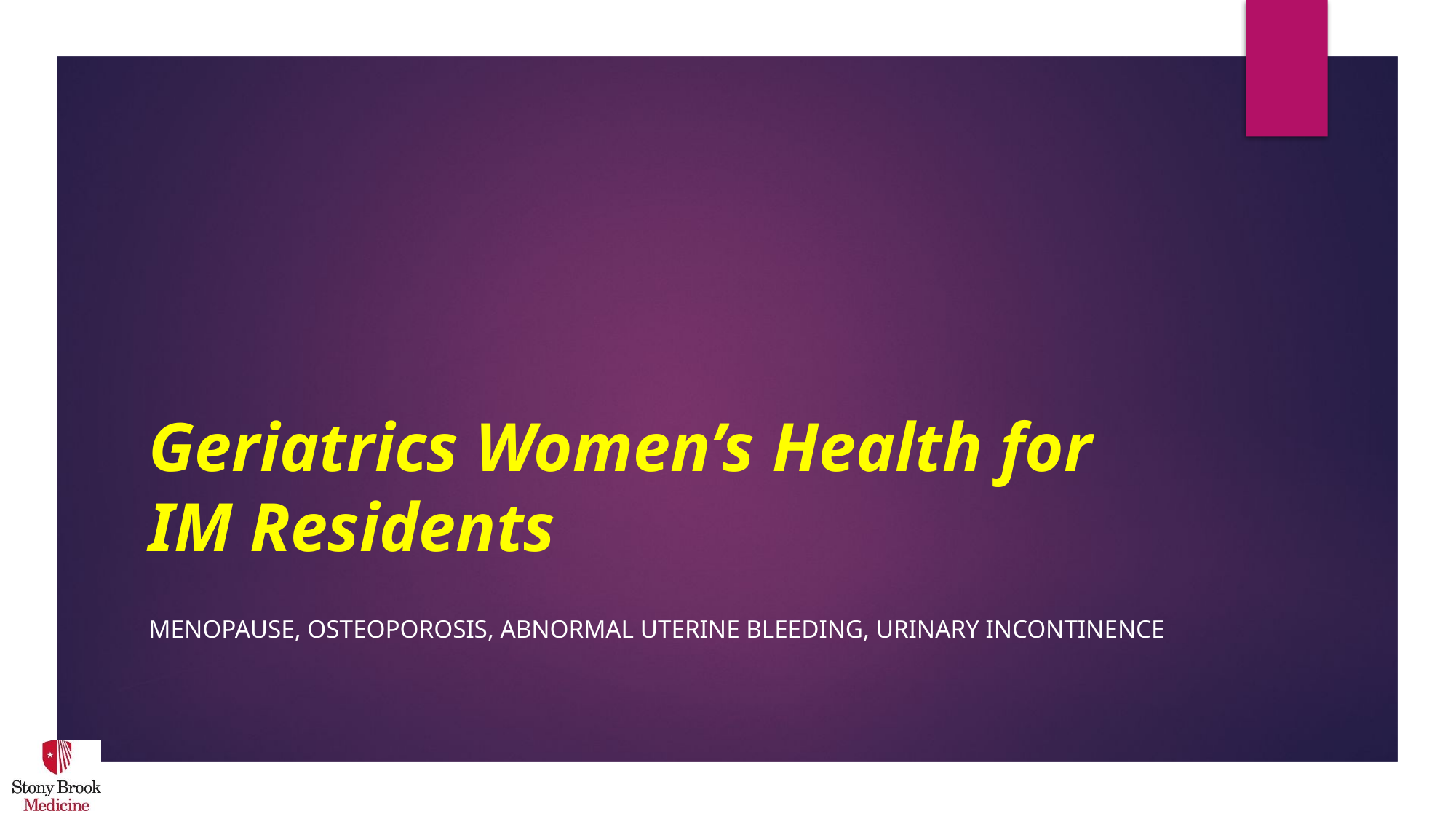

# Geriatrics Women’s Health for IM Residents
Menopause, Osteoporosis, Abnormal uterine bleeding, urinary incontinence

## Slide 2
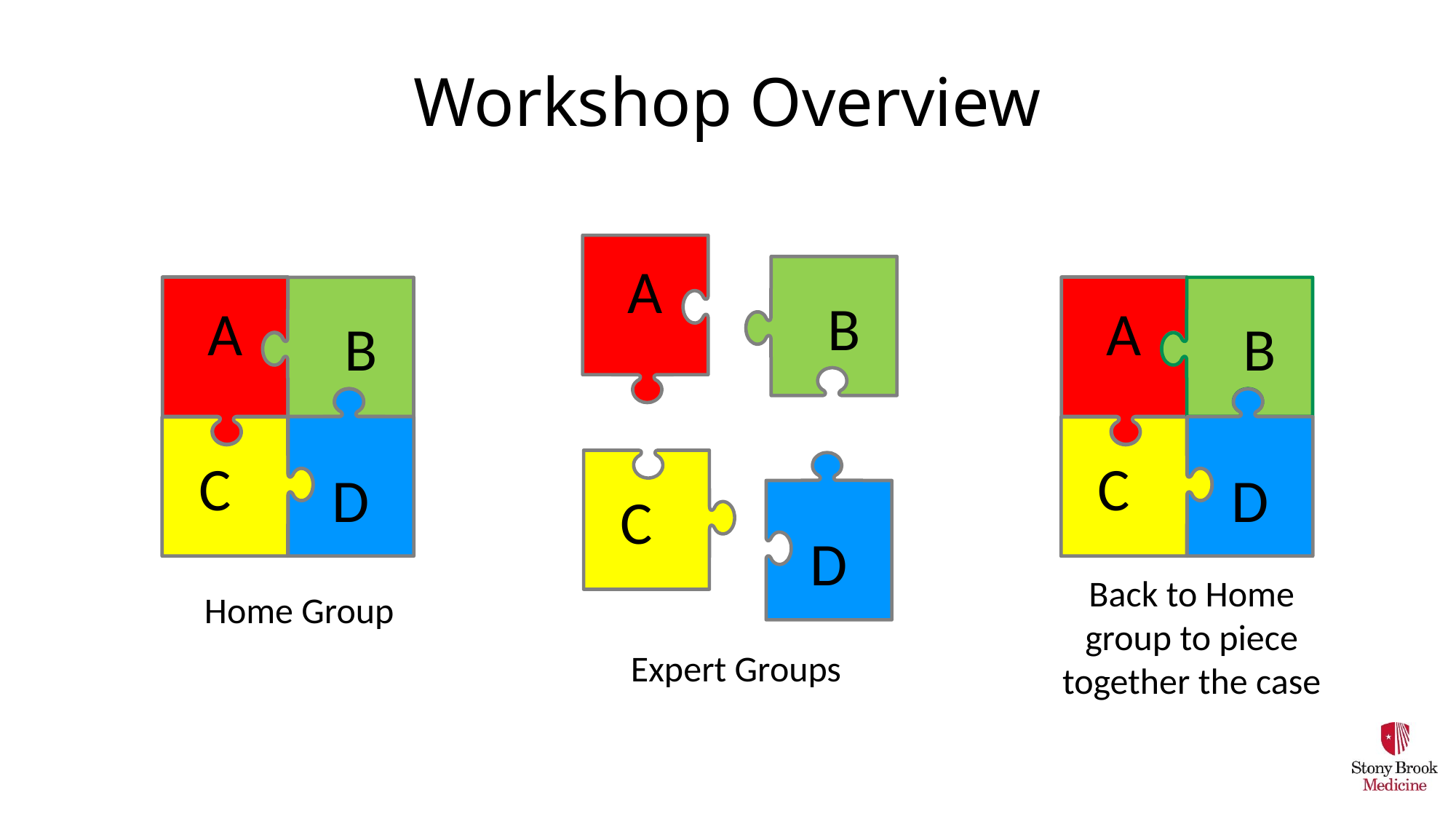

# Workshop Overview
A
B
C
D
Expert Groups
A
A
B
D
C
Back to Home group to piece together the case
B
D
C
Home Group

## Slide 3
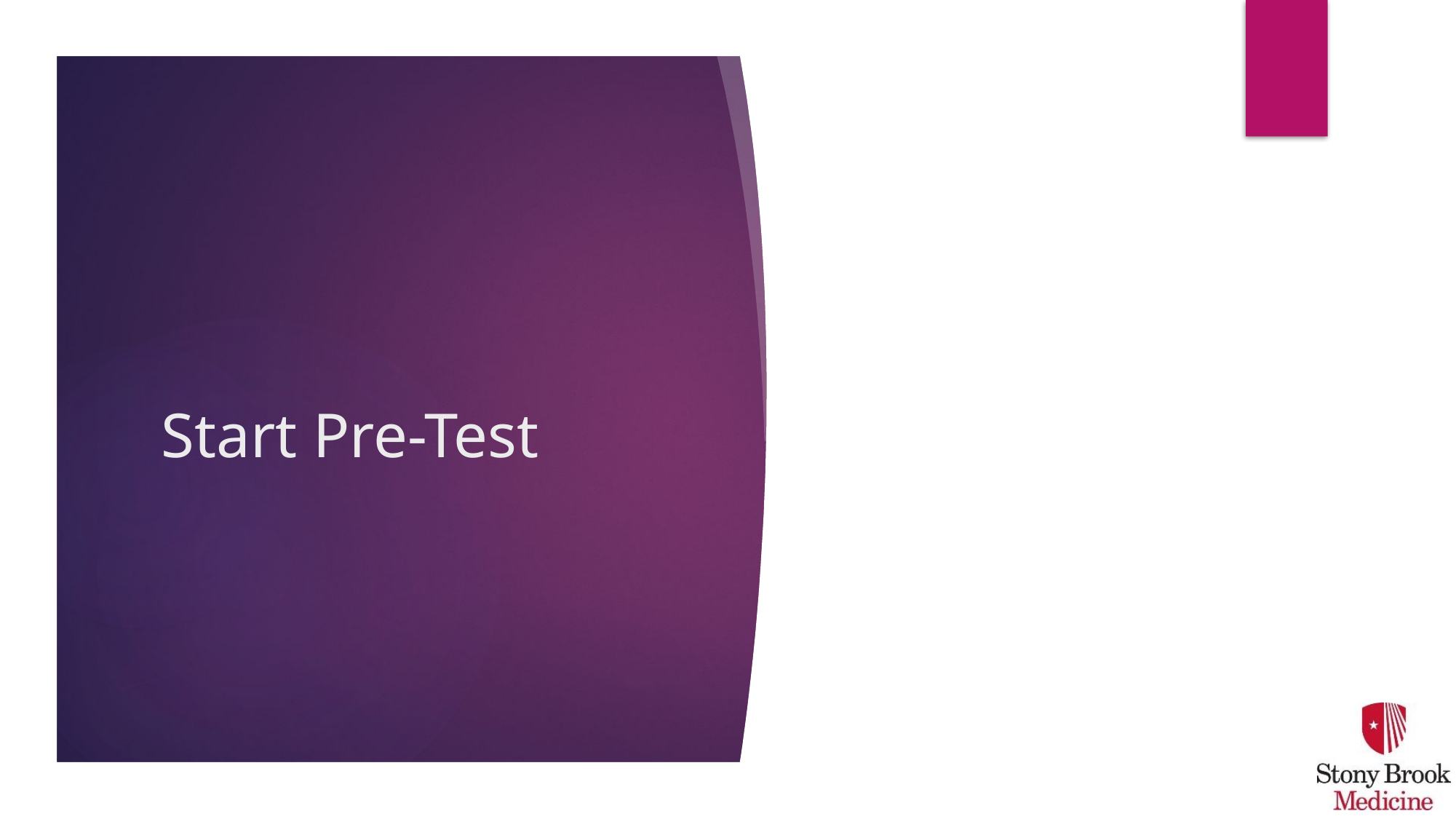

# Start Pre-Test

## Slide 4
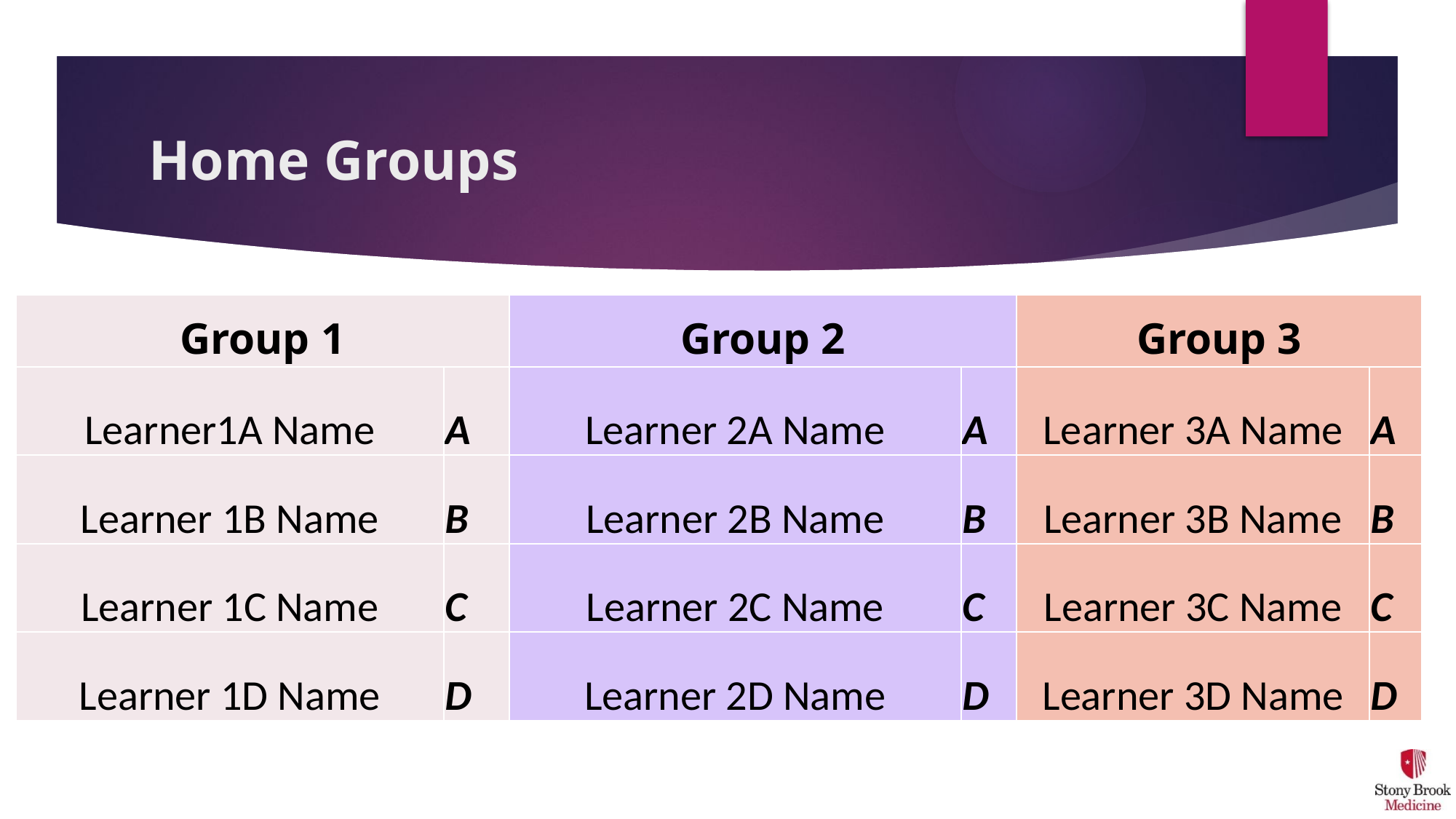

# Home Groups
| Group 1 | | Group 2 | | Group 3 | |
| --- | --- | --- | --- | --- | --- |
| Learner1A Name | A | Learner 2A Name | A | Learner 3A Name | A |
| Learner 1B Name | B | Learner 2B Name | B | Learner 3B Name | B |
| Learner 1C Name | C | Learner 2C Name | C | Learner 3C Name | C |
| Learner 1D Name | D | Learner 2D Name | D | Learner 3D Name | D |

## Slide 5
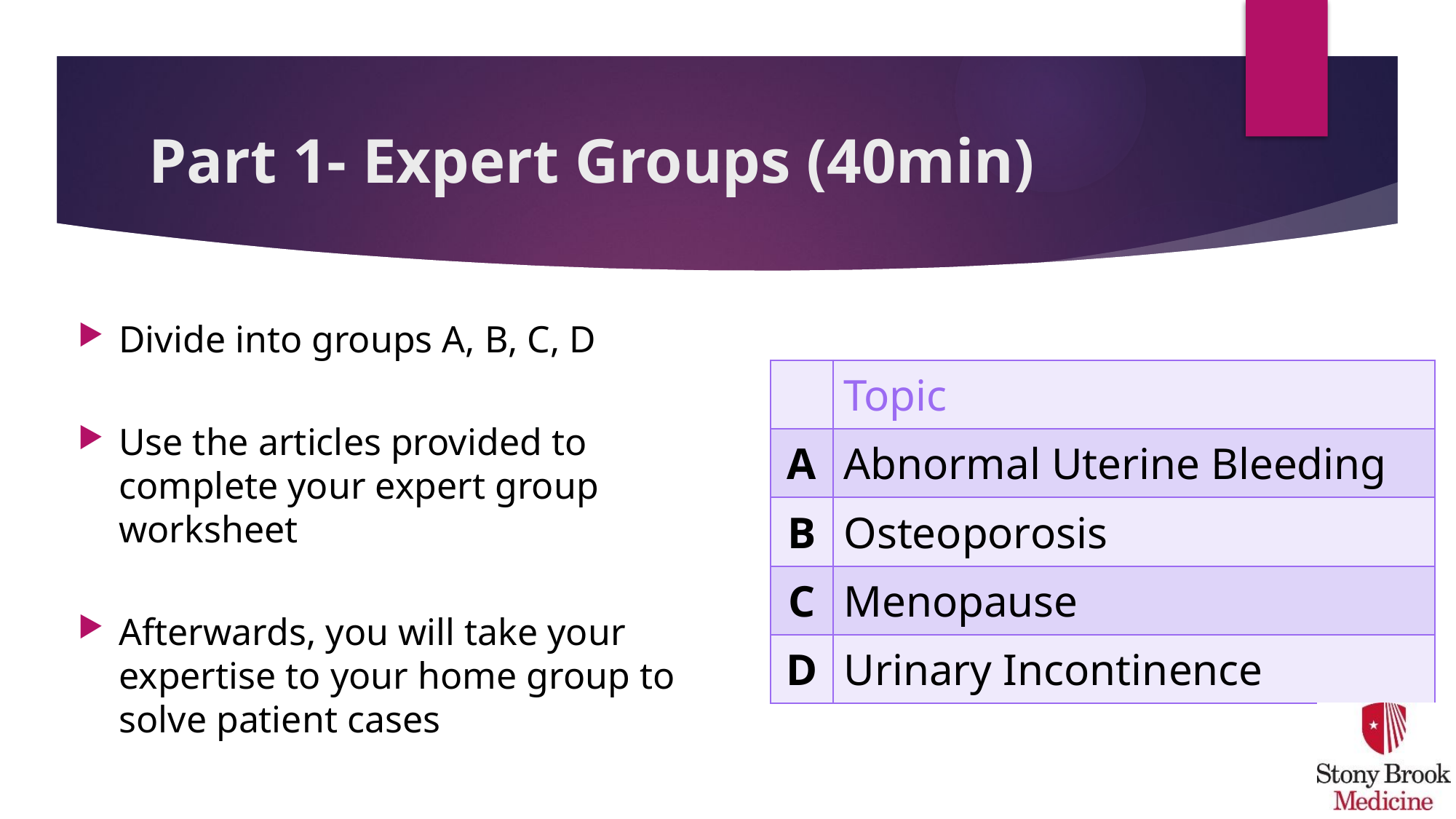

# Part 1- Expert Groups (40min)
Divide into groups A, B, C, D
Use the articles provided to complete your expert group worksheet
Afterwards, you will take your expertise to your home group to solve patient cases
| | Topic |
| --- | --- |
| A | Abnormal Uterine Bleeding |
| B | Osteoporosis |
| C | Menopause |
| D | Urinary Incontinence |

## Slide 6
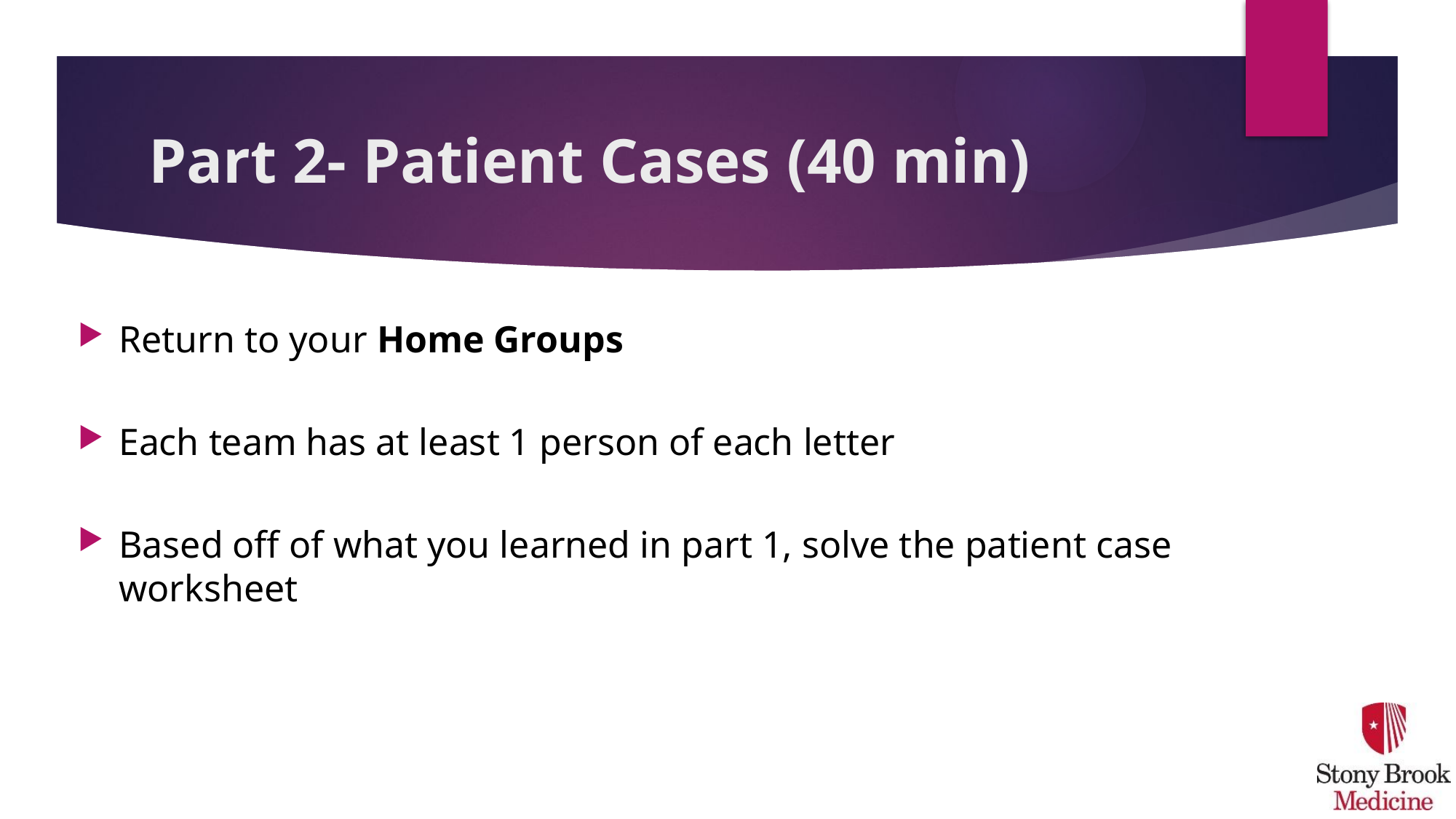

# Part 2- Patient Cases (40 min)
Return to your Home Groups
Each team has at least 1 person of each letter
Based off of what you learned in part 1, solve the patient case worksheet
A
B
D

## Slide 7
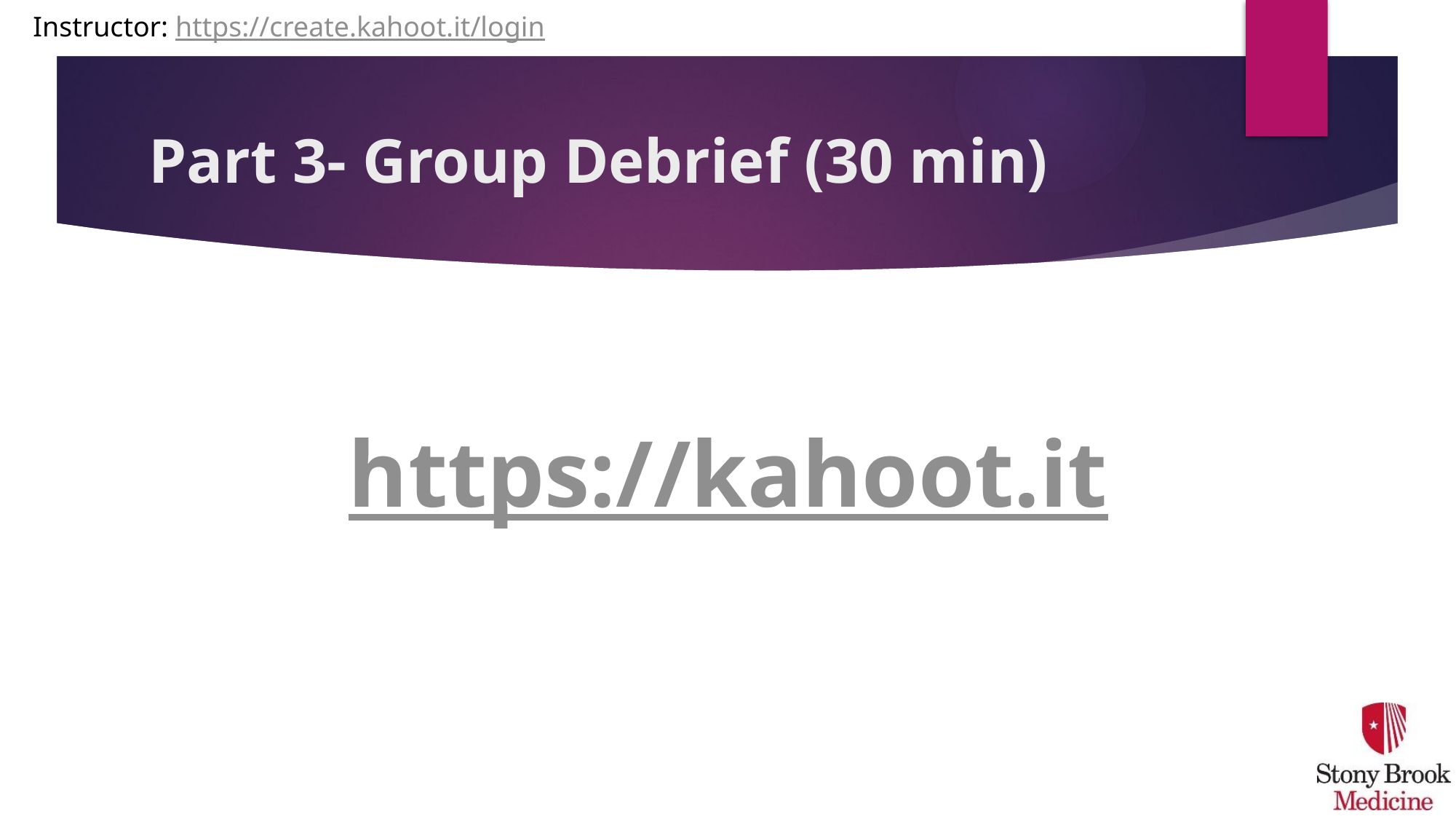

Instructor: https://create.kahoot.it/login
# Part 3- Group Debrief (30 min)
https://kahoot.it

## Slide 8
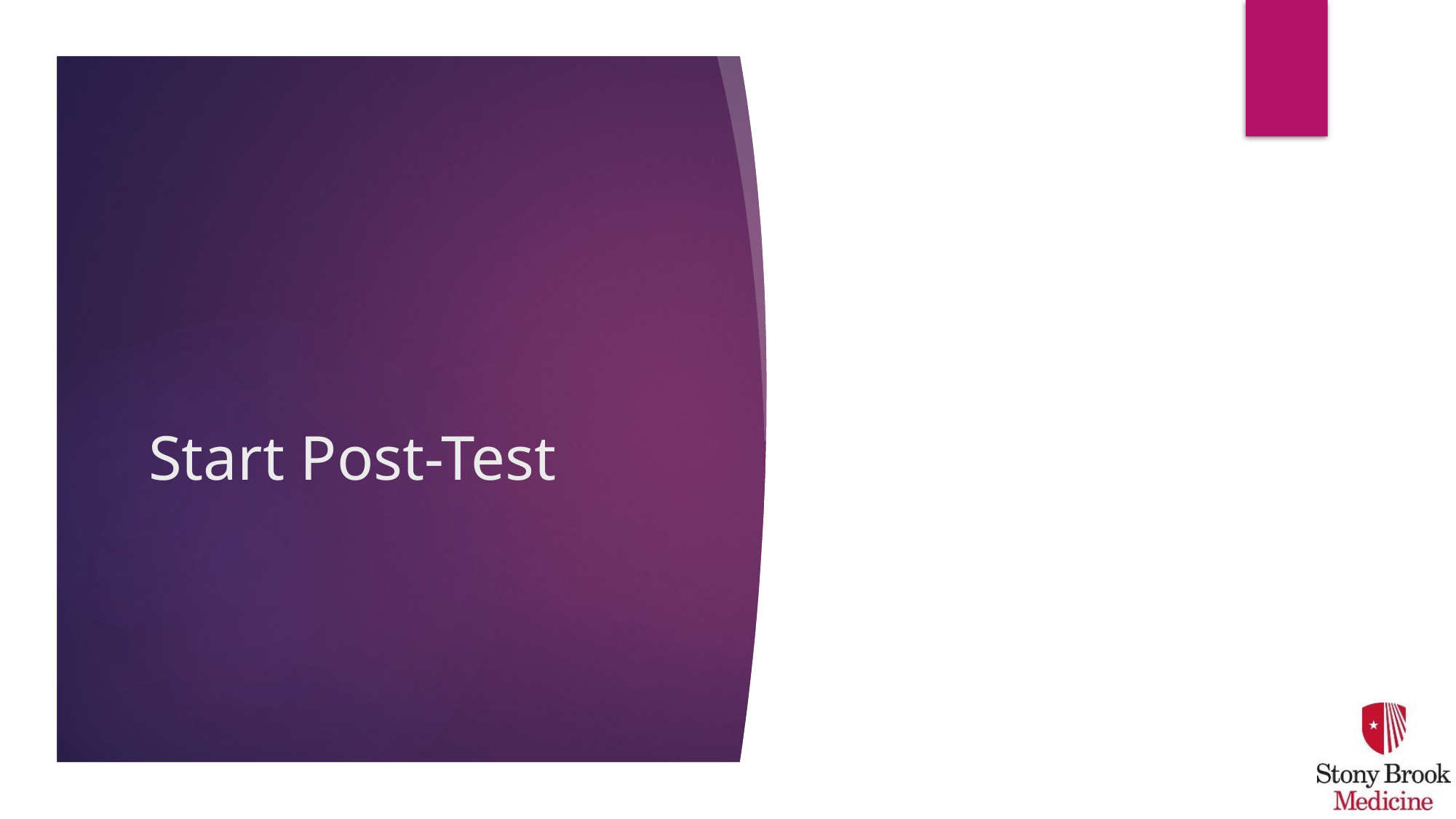

# Start Post-Test
