# Supplementary material for: Using the Jigsaw Teaching Method to Enhance Internal Medicine Residents' Knowledge and Attitudes in Managing Geriatric Women's Health
Source: MedEdPORTAL. 2020 Oct 23;16:11003. doi: 10.15766/mep_2374-8265.11003 (PMC7586752; doi:10.15766/mep_2374-8265.11003)
Supplement: Supplementary file 1 — Expert Group Reading Materials.docxStudent Worksheet-Group A AUB.docxStudent Worksheet-Group B Osteoporosis.docxStudent Worksheet-Group C Menopause.docxStudent Worksheet-Group D UI.docxStudent Worksheet-Patient Cases.docxFacilitator Guide-Group A AUB.docxFacilitator Guide-Group B Osteoporosis.docxFacilitator Guide-Group C Menopause.docxFacilitator Guide-Group D UI.docxFacilitator Guide-Patient Cases and Debriefing Questions.docxFacilitator Guide Overview and Jigsaw Instructions.docxGeriatric Women's Health for IM Residents.pptxPretest.docxPosttest.docx [file mep_2374-8265.11003-s001.zip › N. Pretest.docx]

1. Age: ____
2. Gender:
   1. Male
   2. Female
3. Ethnicity (Check all that apply)
   1. Asian/Pacific Islander
   2. Caucasian
   3. Black/African American
   4. Hispanic/Latino
   5. Native American
   6. Other
   7. Prefer not to answer
4. Please indicate your PGY year:
   1. PGY1
   2. PGY2
   3. PGY3
   4. PGY4
5. What type of medical school training did you complete?
   1. Allopathic Medical School (MD)
   2. Osteopathic Medical School (DO)
   3. Other: _______
6. Where is your continuity clinic?
   1. VA
   2. Tech park
7. Have you ever had Women’s Health training in any of the following venues? (check all that apply)
   1. Undergraduate
   2. Medical school
   3. Graduate school
   4. Residency
   5. Self-study
   6. None
   7. Other (please specify): _______________________________________
8. How confident are you in counseling a patient on Women’s Health issues?
   1. Not confident
   2. Somewhat unconfident
   3. Neutral
   4. Somewhat confident
   5. Confident
9. How likely are you to counsel patients on Women’s Health in the outpatient setting in the next three months?
   1. Very unlikely
   2. Unlikely
   3. Not sure
   4. Likely
   5. Very likely

**Please choose the best answer for the following questions.**

1. 51 year old female presents to you with complaints of hot flashes and vaginal dryness. Her last menstrual period was 13 months ago. She would like to discuss treatment options for her symptoms. You would discuss all the following **EXCEPT**…
   1. Explain that her symptoms are likely due to menopause
   2. Lifestyle modifications including layer clothes, using fans and drinking cold water
   3. Use water-based lubricants for vaginal symptoms
   4. Hormone replacement therapy
   5. SSRI or SNRI
   6. Check thyroid function tests
   7. Evaluate her cardiac risk before starting HRT
   8. Evaluate her breast cancer risk before starting HRT
2. Which of the following is **true**?
   1. Premature menopause is defined as cessation of menses for 12 months or more in patients less than 40 years old
   2. Menopausal symptoms include vasomotor symptoms, sexual dysfunction, and osteoporosis
   3. Hormone replacement therapy with estrogen + progesterone is not effective CAD prevention
   4. HRT increases the risk of breast cancer
   5. All of the above
   6. None of the above
3. Your patient is experiencing menopause symptoms but does not want to start hormone replacement therapy due to fear of side effects. What can you recommend first?
   1. Start clonidine
   2. Start SSRI
   3. Start gabapentin as evening dose
   4. No additional medication
   5. Recommend black cohosh
4. A 68 year old female with a history of smoking, CKD3 and achalasia presents for follow up. She recently had a DEXA scan with a T-score -3.2 at the lumbar spine. In regards to her bone health, you would recommend all the following **EXCEPT**…
   1. Emphasize daily goals of calcium and vitamin D intake
   2. Increase weight bearing exercises, including walking and weightlifting
   3. Start a bisphosphonate
   4. Start denosumab
   5. Smoking cessation
   6. A, B, and E because the patient only has osteopenia
5. For which group of patients is DEXA scan indicated?
   1. All women 50 and older
   2. All women age 65 and older
   3. All women who smoke
   4. All women over age of 75
6. National Osteoporosis Foundation recommends that individuals 50 years and older with DEXA T score -1.0 and -2.5 (osteopenia) should be treated if:
   1. 10 year risk of hip fracture is 3% or greater
   2. 10 year risk of any major osteoporosis-related fracture is 20% or greater
   3. 10 year risk of hip fracture is 20% or greater
   4. All osteopenia patients should be treated
   5. A and B
7. A 45 year old mother presents with complaints of leaking urine with sneezing and coughing. She denies any dysuria, vaginal discomfort, or vaginal discharge. She also has constant urge to urinate and sometimes cannot make it to the bathroom. She 3 spontaneous vaginal births and is a current smoker. What type of urinary incontinence does she have?
   1. Stress incontinence
   2. Urge incontinence
   3. Mixed incontinence
   4. Overflow incontinence
8. What are treatment options for patients who suffer from urinary incontinence?
   1. Weight loss in obese women
   2. Dietary changes, including reduction in alcoholic, caffeinated and carbonated beverages, and amount of liquids
   3. Address constipation
   4. Smoking cessation
   5. Kegel exercises
   6. All of the above
9. A 56-year-old post-menopausal women with HTN and diabetes presents increased urge to urinate and vaginal itchiness. Urinalysis is negative for infection and pelvic exam is normal. Which of the following therapies would you recommend for her symptoms?
   1. Muscarinic agonists
   2. Muscarinic antagonists
   3. Alpha blockers
   4. 5 alpha-reductase inhibitors
   5. Ace inhibitors
10. A 35 year old woman with HTN, obesity, and epilepsy on valproate presents to your clinic for irregular periods. A year ago, her periods were every 27 days but recently her periods come every 40 days. Family history notable for a grandmother who had bleeding problems. To evaluate her symptoms you would do all of the following EXCEPT…
    1. Obtain a detailed gynecology and obstetrical history
    2. Perform a pelvic exam
    3. Order a pregnancy test
    4. Check TSH, prolactin level
    5. Check CBC, PT, PTT, INR
    6. CT of abdomen and pelvis without contrast
    7. I would order all of these tests
11. A 46 y/o female breast cancer survivor presents with heavy periods. Workup is negative for coagulopathy or thyroid dysfunction. Transvaginal US is unrevealing. Endometrial biopsy is negative for malignancy. You may offer all the following therapies **EXCEPT**…
    1. NSAIDs
    2. Progestin-only pill
    3. Tranexamic acid
    4. Hysterectomy
    5. I would offer all the therapies above
12. Risk factors for endometrial cancer include…
    1. Exposure to unopposed estrogen
    2. Obesity
    3. Older than 35 years old
    4. Diabetes
    5. Family history of colon cancer
    6. Infertility
    7. History of tamoxifen use
    8. A, B, C only
    9. All of the above
    10. None of the above
